# Selected Class of Enamides Bearing Nitro Functionality as Dual-Acting with Highly Selective Monoamine Oxidase-B and BACE1 Inhibitors

**DOI:** 10.3390/molecules26196004

**Published:** 2021-10-03

**Authors:** Anusree Venkidath, Jong Min Oh, Sanal Dev, Elham Amin, Shebina P. Rasheed, Ajeesh Vengamthodi, Nicola Gambacorta, Ahmed Khames, Mohamed A. Abdelgawad, Ginson George, Orazio Nicolotti, Hoon Kim, Bijo Mathew

**Affiliations:** 1 Centre for Experimental Drug Design and Development, Department of Pharmaceutical Chemistry, Al-Shifa College of Pharmacy, Perinthalmanna 679325, India; anusreekousthubham96@gmail.com (A.V.); shebinaniz@gmail.com (S.P.R.); ajeeshvengan@hotmail.com (A.V.); 2 Department of Pharmacy, and Research Institute of Life Pharmaceutical Sciences, Sunchon National University, Suncheon 57922, Korea; 1205027@s.scnu.ac.kr; 3 Department of Medicinal Chemistry and Pharmacognosy, College of Pharmacy, Qassim University, Buraidah 52571, Saudi Arabia; elham_bns@yahoo.com; 4 Department of Pharmacognosy, Faculty of Pharmacy, Beni-Suef University, Beni-Suef 62514, Egypt; 5 Dipartimento di Farmacia-Scienze del Farmaco, Università Degli Studi di Bari “Aldo Moro”, Via E. Orabona, 4, I-70125 Bari, Italy; nicola.gambacorta1@uniba.it (N.G.); orazio.nicolotti@uniba.it (O.N.); 6 Department of Pharmaceutics and Industrial Pharmacy, College of Pharmacy, Taif University, P.O. Box 11099, Taif 21944, Saudi Arabia; a.khamies@tu.edu.sa; 7 Department of Pharmaceutical Chemistry, College of Pharmacy, Jouf University, Sakaka 72341, Saudi Arabia; mhmdgwd@ju.edu.sa; 8 Department of Pharmaceutical Chemistry, Amrita School of Pharmacy, Amrita Vishwa Vidyapeetham, AIMS Health Sciences Campus, Kochi 682041, India; ginsongeorge239@gmail.com

**Keywords:** monoamine oxidase, β-secretase, nitro group-bearing enamides, potent reversible inhibitor, dual-acting inhibitor, molecular docking

## Abstract

A small series of nitro group-bearing enamides was designed, synthesized (**NEA1**–**NEA5**), and evaluated for their inhibitory profiles of monoamine oxidases (MAOs) and β-site amyloid precursor protein cleaving enzyme 1 (β-secretase, BACE1). Compounds **NEA3** and **NEA1** exhibited a more potent MAO-B inhibition (IC_50_ value = 0.0092 and 0.016 µM, respectively) than the standards (IC_50_ value = 0.11 and 0.14 µM, respectively, for lazabemide and pargyline). Moreover, **NEA3** and **NEA1** showed greater selectivity index (SI) values toward MAO-B over MAO-A (SI of >1652.2 and >2500.0, respectively). The inhibition and kinetics studies suggested that **NEA3** and **NEA1** are reversible and competitive inhibitors with K_i_ values of 0.013 ± 0.005 and 0.0049 ± 0.0002 µM, respectively, for MAO-B. In addition, both **NEA3** and **NEA1** showed efficient BACE1 inhibitions with IC_50_ values of 8.02 ± 0.13 and 8.21 ± 0.03 µM better than the standard quercetin value (13.40 ± 0.04 µM). The parallel artificial membrane permeability assay (PAMPA) method demonstrated that all the synthesized derivatives can cross the blood–brain barrier (BBB) successfully. Docking analyses were performed by employing an induced-fit docking approach in the GLIDE module of Schrodinger, and the results were in agreement with their in vitro inhibitory activities. The present study resulted in the discovery of potent dual inhibitors toward MAO-B and BACE1, and these lead compounds can be fruitfully explored for the generation of newer, clinically active agents for the treatment of neurodegenerative disorders.

## 1. Introduction

The development of a new class of molecules for the complex pathology of neurodegenerative disorders, like Alzheimer’s disease (AD) and Parkinson’s disease (PD), is one of the most complicated zones in medicinal chemistry [1]. Multi-target-directed ligands (MTDLs) have led to a new paradigm that has emerged in recent times, in which the newly designed molecular scaffold is able to bind to different types of biologic targets that are interconnected with similar biochemical pathways [2,3]. The MTDLs design strategy includes the combination of two pharmacologically active molecules into a single framework, or keeping the most active functional moieties of different molecules in a single hybrid molecule [4].

The oxidative deamination of monoamine-related neurotransmitters and their regulation in the central and peripheral systems are controlled by flavin-dependent monoamine oxidases (MAOs) isoenzymes, such as MAO-A and MAO-B [5,6]. Selective inhibitions by MAO-B inhibitors are considered to be a promising neuronal pharmacotherapy for AD and PD [7]. The oxidative stress provoked by the metabolism of MAO-B leads to the cognitive destruction and aggregation of neurofibrillary tangles in PD patients [8,9]. Recently, many scaffolds, like chalcones, coumarins, chromones, pyrazolines, quinazolines, isatins, and thiazolidinones, which are derivatives of FDA approved MAO-B inhibitors, showed selective, reversible, and competitive types of MAO-B inhibition [10,11,12,13,14,15,16,17,18].

The presence of α,β-unsaturated ketone, carboxamide, multi-conjugated ketone, and olefinic linkage contribute to the versatility of the pharmacophore features of selective MAO-B inhibitors [19,20,21,22,23,24]. Recently, our group developed a new class of enamide-based MAO-B inhibitors by combining an olefinic linkage to the amide functional group. The resulting enamide linker with nitrophenyl hydrophobic candidate showed potent and selective MAO-B inhibition. The study documented that the presence of an electron-withdrawing nitro group has better binding affinity than electron-donating groups. Compound N-(3-nitrophenyl)prop-2 enamide (**AD9**) was an effective competitive inhibitor of MAO-B with Ki value of 0.039 ± 0.005 µM [25]. In addition, numerous reports suggested that nitro groups are versatile electron-attracting functional groups, which are used in many of the FDA-approved drugs. This unique functional group can act as a proton acceptor, which can contribute to hydrogen-bonding interactions within the catalytic site of a variety of enzyme targets [26,27]. Prompted by this observation, we expanded our examination of structure activity relationships (SARs) of enamide-based MAO-B inhibitors by keeping nitrophenyl functionalities with other selected classes of enamide-based compounds.

The inhibition of the β-site amyloid precursor protein-cleaving enzyme-1 (BACE1) is one of the other attractive objectives when treating AD [28]. BACE1 inhibitors that are currently in different stages of clinical trials, such as Verubecestat, Elenbecestat, and Atabecestat, have an amide functional moiety as the crucial pharmacophore feature [29]. In this research, we focused our attention on the development of a small library of nitro-substituted enamide derivatives with dual-acting MAO-B and BACE1 inhibitors. The design and development of MTDLs has become authoritative in the selection of the starting scaffolds to develop a multifunctional molecular framework. The objective of the work is the synthesis and characterization of a selected class of nitro-bearing enamides. All the molecules were further subjected to enzyme inhibition studies against MAO-A, MAO-B, and BACE1. The topmost active compounds were exploited for their kinetics, reversibility properties, parallel artificial membrane permeability assay (PAMPA), and docking analyses.

## 2. Results and Discussion

### 2.1. Chemistry

**Scheme 1:**
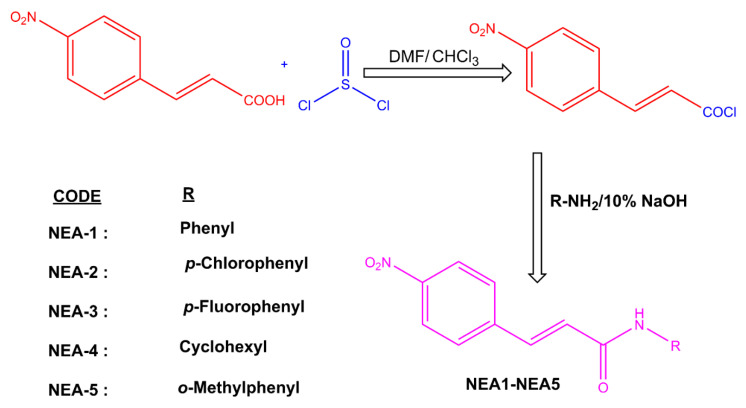
Synthesis of enamides.

The selected class of compound was synthesized by the following approach (Scheme 1). The spectral data are provided in the Supplementary Materials. The structures of the compounds were verified with reference to previously published literature [30,31].

### 2.2. Studies of MAOs and BACE1 Inhibitors

All of the screened compounds effectively inhibited MAO-B with residual activities of <50% at 10 μM. However, these compounds showed a greater residual activity toward MAO-A (>83.5% at 10 μM, except **NEA-3**) over MAO-B, indicating that these compounds possess greater inhibitory potential for MAO-B (Table 1). Compounds **NEA3** and **NEA1** exhibited the most potential activity toward MAO-B (IC_50_ value = 0.0092 and 0.016 μM, respectively) (Table 1). Unsubstituted enamide (**NEA1**) exerted an IC_50_ of 0.016 μM. Substitution of numerous functionalities exerted a varied MAO-B inhibitory profile. For instance, a strong electron-withdrawing group (-F atom) at the *para* position resulted in the formation of a more potent component (**NEA3**; IC_50_ = 0.0092 μM) than the parent (**NEA3**). Interestingly, an electron-donating substituent (**NEA5** with methyl functionalities at the *ortho* side) also resulted in the enhancement of MAO-B inhibition. It is noteworthy that the -Cl atom on the *para* position in **NEA2** resulted in the weakening of MAO-B inhibitory activity. This needs to be investigated further. In these derivatives, MAO-B inhibitory activities increased with the presence of *para*-F (**NEA3**) > -H (**NEA1**) > *ortho*-CH_3_ (**NEA5**) > *para*-Cl (**NEA2**) (Table 1). The selectivity for MAO-B over MAO-A was also observed in terms of the selectivity index (SI), where **NEA1** and **NEA3** exerted high SI values of 2500 and 1652.2, respectively (Table 1). In addition, as represented in Table 1, **NEA1**, **NEA3**, and **NEA5** exerted great inhibitory potentials toward BACE1 (IC_50_ = 8.21, 8.02, and 17.7 μM, respectively). The IC_50_ values of **NEA1** and **NEA3** were lower than the reference quercetin (IC_50_ = 13.4 μM). Quercetin has been used as a reference for BACE1 inhibition, and the IC_50_ values ranged between 8.65 and 20.18 μM [32,33,34]. On the other hand, all of the compounds weakly inhibited acetylcholinesterase and butyrylcholinesterase, with the residual activities of >76.5% at 10 μM.

Recently, our group developed some trimethoxy halogenated chalcones as dual-acting MAO-B and BACE1 inhibitors that exhibited activities in the range of 0.84 to 4.17 µM and 13.6 to 19.8 µM, respectively [35]. In another study, phytochemicals from *Rauwolfia serpentina* roots were evaluated for their MTDL efficiencies, while indole alkaloids (reserpine and ajmalicine) were evaluated as potential agents for MAO-B and BACE1 inhibition with dose-dependent activities [36]. It is noteworthy that the replacement of chalcones with enamides resulted in the enhancement of overall MAO-B and BACE1 inhibitory activities. Interestingly, the analogues which we tested resulted in potent inhibitory activities in nanomolar ranges towards MAO-B inhibition.

### 2.3. Kinetics

**Figure 1:**
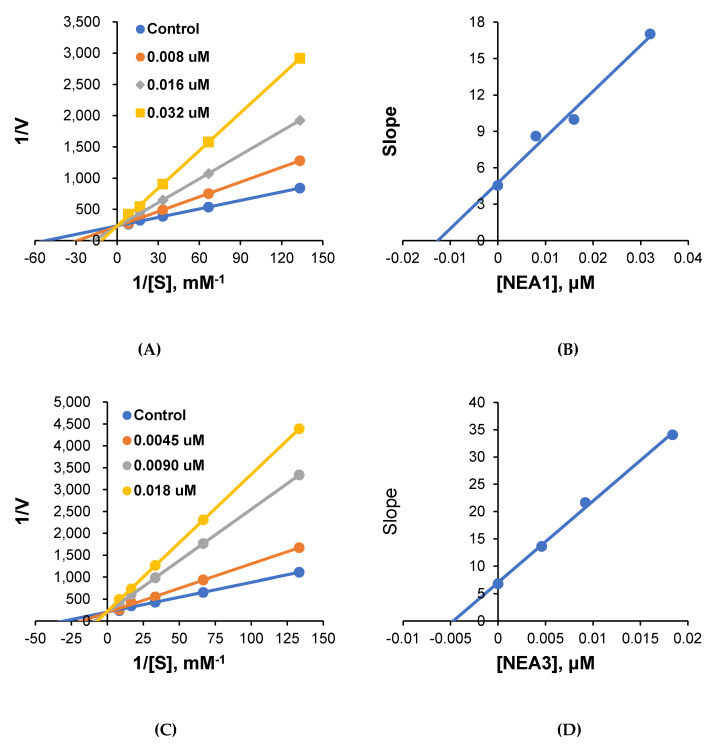
Lineweaver–Burk plots for MAO-B inhibition by **NEA1** and **NEA3** (**A**,**C**), and their respective secondary plots (**B**,**D**) of the slopes vs. inhibitor concentrations.

The most potent compounds, **NEA1** and **NEA3**, were further subjected to enzyme-inhibition kinetics. The obtained results are represented in Figure 1. As represented in Lineweaver–Burk plots, all of the lines converged at the y-intercept (1/V_max_), suggesting the scope of the competitive mode of inhibition by these compounds. The K_i_ values were deduced from the secondary plot, and were found to be 0.013 ± 0.005 and 0.0049 ± 0.0002 μM for **NEA1** and **NEA3**, respectively. These results suggest the competitive mode of inhibition by these compounds at the active site of MAO-B.

### 2.4. Reversibility Studies

**Figure 2:**
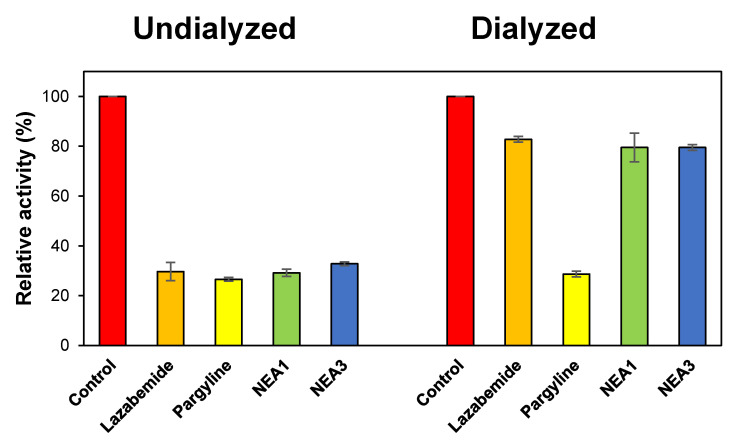
Recoveries of MAO-B inhibitions by **NEA1** and **NEA3** using dialysis experiments. Dialysis was performed for 6 h with a buffer change after 3 h with duplicate experiments.

The reversibility patterns of the potent compounds were analyzed by comparing the relative activities in undialyzed (A_U_) and dialyzed (A_D_) samples at the following concentrations: **NEA1** = 0.032 μM; **NEA3** = 0.020 μM; lazabemide = 0.22 μM; and pargyline = 0.28 μM. Lazabemide is a reversible inhibitor of MAO-B, while pargyline is an irreversible inhibitor of MAO-B; thus, they were selected as reference standards. In the case of **NEA1**, the MAO-B inhibition was recovered from 29.2% (A_U_) to 79.5% (A_D_), while for **NEA3** it went from 32.8% to 79.5% (Figure 2). These recovery values were similar to the reversible reference inhibitor (lazabemide), which went from 29.7% to 82.8%, and could be distinguished to pargyline, an irreversible reference inhibitor, which went from 26.6% to 28.7%. These results indicate that **NEA1** and **NEA3** function as reversible MAO-B inhibitors.

### 2.5. Blood–Brain Barrier (BBB) Permeation Studies

The CNS bioavailability of the molecules was further ascertained by the PAMPA method [37]. Highly effective permeability and high CNS bioavailability were observed for all the enamides tested, with Pe ranges between 13.46 × 10^−6^ and 16.43 × 10^−6^ cm/s (Table 2).

### 2.6. Computational Studies

**Figure 3:**
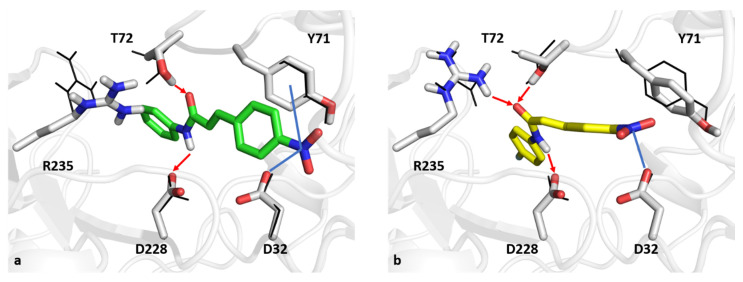
The best-docked poses of compounds **NEA1** (**a**, green sticks) and **NEA3** (**b**, yellow sticks) in a BACE1 binding pocket. Blue lines and red arrows indicate ion-dipole interactions and hydrogen bonds, respectively. Black wireframes report the original pose of BACE1 sidechains in the binding site.

**Figure 4:**
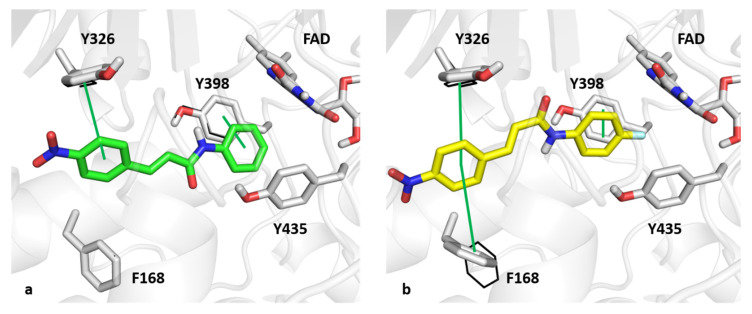
The best-docked poses of compounds **NEA1** (**a**, green sticks) and **NEA3** (**b**, yellow sticks) in a MAO-B binding pocket. Green lines indicate p–p contacts. Black wireframes report the original pose of MAO-B sidechains within the binding site.

In silico analyses were performed at the molecular level to deepen the interactions between compounds **NEA1** and **NEA3** and enzymes BACE1 and MAO-B. Figure 3 and Figure 4 show the results of induced-fit docking.

As far as interactions within BACE1 are concerned, the **NEA1** compound can make hydrogen bonds with T72 and the catalytic D228 with its enamide moiety. Moreover, the *para*-nitro substituent can make an ion-dipole interaction with the catalytic D32 and a dipole interaction with Y71 at S1. On the other hand, the enamide moiety of compound **NEA3** can establish hydrogen bonds with the catalytic D228 and T72 as reported for **NEA1** and, in addition, with R235. Furthermore, the *para*-nitro group can interact through ion-dipole interaction with catalytic D32. For the sake of completeness, docking scores for **NEA1** and **NEA3** were equal to 6.656 and 7.191 kcal/mol, respectively.

As for the MAO-B binding pocket, **NEA1** and **NEA3** assumed a similar binding mode within the MAO-B active site. The *para*-nitro aromatic ring of **NEA1** was able to make p–p contact with the MAO-B selective residue, Y326, and the phenyl ring that resulted was placed in a network of hydrophobic interactions with FAD, Y435, and Y398, the latter also being involved in p–p contact. Regarding the binding mode of **NEA3**, the *para*-nitro aromatic ring that resulted was sandwiched between Y326 and F168. Furthermore, the *para*-fluorine ring was able to interact with Y398 through p–p contact, which resulted in it being trapped in the aromatic cage that Y398, Y435, and FAD formed. Docking score values obtained from simulations of **NEA1** and **NEA3** were found to be −9.977 and −10.453 kcal/mol, respectively.

Computational studies have shed light on the interaction between compounds **NEA1** and **NEA3** toward BACE1 and MAO-B. Interestingly, the induced-fit docking protocol can give a more accurate and detailed description of the binding modes of the two compounds. As far as BACE1 is concerned, the conformation of the two catalytic aspartate residues (D32 and D228) slightly changed for the optimal interaction with the two compounds. In addition, the carbonyl group of the enamides formed a hydrogen bond with the conformation T72 residue located in the FLAP region. Furthermore, R235 was particularly affected by the induced-fit docking, favoring the interaction with the enamide moiety of compound **NEA3**. As for MAO-B, the only residues involved in a change of its conformation state was F168, whose movement allowed the T-shape p–p contact with the *para*-nitro aromatic ring of compound **NEA3**.

## 3. Materials and Methods

### 3.1. Synthesis

The synthesis of the target molecules is depicted in Scheme 1. Initially, 4-nitrocinnamic acid (5 mmol, i.e., 1.0 g) was added into a 100 mL two-neck flask containing 20 mL of chloroform. Four drops of DMF were added slowly to the flask and stirred for 15 min at room temperature (RT). Thionyl chloride (10 mmol, 2 mL) was added and heated to reflux at 50–60 °C for 5 h. After completion of the reaction, the excess thionyl chloride was distilled off to obtain 4-nitrocinnamoyl chloride as a yellow solid. It was then transferred into a 250 mL flask containing a mixture of respective amines (10 mmol) and 50 mL of 10% NaOH. The reaction was stirred at RT until the completion of the reaction was confirmed by TLC. The final compounds were obtained by recrystallization from hot ethanol.

#### 3.1.1. 3-(4-Nitrophenyl)-*N*-phenylacrylamide (**NEA1**)

Whitish-yellow powder; M.P. 210–212 °C; R_f_ value: 0.68 (*n*-hexane ethyl acetate = 2:0.5); IR (ZnSe): 3305 cm^−1^ (N-H stretching in amide), 1655 cm^−1^ (C=O stretching in amide), 1621 cm^−1^ (Ar-CH=CHR), 1592 cm^−1^ (Ar C-C stretching), 652 cm^−1^ (=CH out of plane in trans RHC=CHR); ^1^H NMR (500 MHz) DMSO-*d*_6_ δ ppm: 10.35 (s, 1H, NH), 8.30 (d, *J* = 5, 1H, CH), 7.91–7.81 (m, 2H), 7.01–7.91 (m, 9H, Ar H); ^13^C NMR (125 MHz) DMSO-*d*_6_: 119.28, 123.58, 124.09, 126.55, 128.66, 128.78, 137.61, 138.98, 141.25, 147.57, 162.75; ESI-MS (*m*/*z*): 269.0138 [M + 1].

#### 3.1.2. *N*-(4-Chlorophenyl)-3-(4-nitrophenyl)acrylamide (**NEA2**)

Brownish-yellow powder; M.P. 224–226 °C; Rf value: 0.8 (*n*-hexane ethyl acetate = 2:0.5); IR (ZnSe): 3377 cm^−1^ (N-H stretching in amide), 1608 cm^−1^ (C=O stretching in amide), 815 cm^−1^ (C-Cl stretching), 1490 cm^−1^ (Ar C-C stretching), 692 cm^−1^ (=CH out of plane in trans RHC=CHR); ^1^H NMR (500 MHz) DMSO-*d*_6_ δ ppm: 10.49 (s, 1H, NH), 8.29–8.30 (d, *J* = 5 2H, CH), 6.98–7.91 (m, 8H, Ar H); ^13^C NMR (125 MHz) DMSO-*d*_6_: 24.48, 25.20, 32.36, 47.72, 124.09, 126.84, 128.50, 136.13, 141.65, 147.43, 163.27; ESI-MS (*m*/*z*): 301.1145 [M + 1].

#### 3.1.3. *N*-(4-Fluorophenyl)-3-(4-nitrophenyl)acrylamide (**NEA3**)

Whitish-yellow powder; M.P. 208–210 °C; Rf value: 0.61 (*n*-hexane ethyl acetate = 2:0.5); IR (ZnSe): 3289 cm^−1^ (N-H stretching in amide), 1606 cm^−1^ (C=O stretching in amide), 1402 cm^−1^ (Ar C-C stretching), 958 cm^−1^ (C-F stretching), 706 cm^−1^ (=CH out of plane in trans RHC=CHR); ^1^H NMR (125 MHz) DMSO-*d*_6_ δ ppm: 10.49 (s, 1H, NH), 8.30–8.28 (d, *J* = 10 2H, CH), 6.97–7.91 (m, 8H, Ar H); ^13^C NMR (125 MHz) DMSO-*d*_6_: 115.39, 120.95, 124.01, 126.25, 128.61, 135.31, 137.61, 141.12, 147.51, 157.13, 159.04, 162.57; ESI-MS (*m*/*z*): 287.0211 [M + 1].

#### 3.1.4. *N*-Cyclohexyl-3-(4-nitrophenyl)acrylamide (**NEA4**)

Whitish-yellow powder; M.P. 166–168 °C; Rf value: 0.49 (*n*-hexane ethyl acetate = 2:0.5); IR (ZnSe): 3370 cm^−1^ (N-H stretching in amide), 2935 cm^−1^ (C-H stretching), 1618 cm^−1^ (C=O stretching in amide), 725 cm^−1^ (=CH out of plane in trans RHC=CHR); ^1^H NMR (500MHz) DMSO-*d*_6_ δ ppm: 8.27–8.25 (d, *J* = 10, 2H, CH), 8.14 (1H, NH), 6.81–8.12 (m, 4H, Ar-H), 3.65–3.69 (m,1H, cyclohexyl), 1.18–1.82 (m, 10H, cyclohexyl); ^13^C NMR (125 MHz) DMSO-*d*_6_: 24.48, 25.20, 32.36, 47.72, 124.09, 126.84, 128.50, 136.13, 141.65, 147.43, 163.27; ESI-MS (*m*/*z*): 275.0888 [M + 1].

#### 3.1.5. 3-(4-Nitrophenyl)-*N*-(*O*-tolyl)acrylamide (**NEA5**)

Whitish-yellow powder; M.P. 200–203 °C; Rf value: 0.58 (*n*-hexane ethyl acetate = 2:0.5); IR (ZnSe): 3270 cm^−1^ (N-H stretching in amide), 1615 cm^−1^ (C=O stretching in amide), 2918 cm^−1^ (C-H stretching), 1450 cm^−1^ (Ar C-C stretching), 720 cm^−1^ (=CH out of plane in trans RHC=CHR); ^1^H NMR (500 MHz) DMSO-*d*_6_ δ ppm: 9.61 (s,1H, NH), 8.30–8.29 (d, *J* = 10 2H, CH), 7.09–7.91 (m, 8H, Ar-H), 2.26 (s, 3H, -CH_3_); ^13^C NMR (125 MHz) DMSO-*d*_6_: 17.82, 124.05, 124.22, 125.10, 125.92, 126.52, 128.62, 130.28, 130.94, 136.05, 137.46, 141.33, 147.51, 162.82; ESI-MS (*m*/*z*): 283.0648 [M + 1].

### 3.2. Enzyme Inhibition Studies

MAO inhibitory assay was assessed in accordance with the previously standardized methods of assessing recombinant MAO-A and MAO-B by using the substrates (0.06 mM kynuramine and 0.3 mM benzylamine, respectively) [38,39,40]. Toloxatone/clorgyline and lazabemide/pargyline were used as reference standards for MAO-A and MAO-B, respectively. The K_m_ of benzylamine for MAO-B was 0.027 mM. The BACE1 activity was measured using a commercially available assay kit (β-secretase, CS0010). An excitation wavelength of 320 nm and an emission wavelength of 405 nm were used in a fluorescence spectrometer (FS-2, Scinco, Seoul, Korea) [41]. Quercetin was used as a reference compound for BACE1. The enzymes and chemicals were purchased from Sigma-Aldrich (St. Louis, MO, USA).

### 3.3. Enzyme Inhibition and Kinetic Studies

Initially, the inhibitory activities of the synthesized compounds were identified at a concentration of 10 μM against MAO-A, MAO-B, and BACE1. Further, the IC_50_ of the compounds which had less than 50% of residual activity were identified for each of the respective enzymes. The obtained IC_50_ values thus compared were used to deduce the SI values. Five different concentrations of the substrates were used in the kinetic experiments. The enzyme kinetics and inhibition patterns were analyzed using the Lineweaver–Burk plots and their secondary plots, under three inhibitor concentrations [42].

### 3.4. Inhibitor-Reversibility Analysis

The reversibility of MAO-B inhibition by **NEA1** and **NEA3** was evaluated using a standardized dialysis method [26]. Initially, the enzyme was preincubated with the screened compounds for 30 min at ~2 × IC_50_ (i.e., 0.032 and 0.020 μM, respectively). Lazabemide (0.22 μM) and pargyline (0.28 μM) were used as reference standards for reversible and irreversible MAO-B inhibitors, respectively. The activities in dialyzed (A_D_) and undialyzed (A_U_) samples were compared and reversibility patterns were determined [43].

### 3.5. Computational Studies

Computational studies of compounds **NEA1** and **NEA3** were performed on X-ray structures of BACE1 (PDB ID: 3TPP) and MAO-B (PDB ID: 2V5Z), whose crystals were retrieved from the Protein Data Bank [44,45]. Geometrical optimization and energetic minimization steps were carried out on proteins by using the Protein Preparation Tools available on the Schrodinger Suite [46]. Ligands were prepared with the Ligprep Tool. The enclosing boxes were centered on cognate ligands [47]. The induced-fit protocol was used by employing GLIDE software with the OLPS3 force field, for the purpose of inspecting the binding mode of ligands as well as the conformational changes in the targets’ sidechains, thus increasing the accuracy and reliability of the results with respect to the standard docking protocols, for which such structural changes are not detectable. Sidechain refinement was carried out on residues within 6 Å of ligand poses together with Glide SP redocking of each protein–ligand complex structure within 30.0 kcal/mol of the lowest energy [48,49,50,51].

## 4. Conclusions

A selected class of enamides bearing nitro pharmacophore was designed, synthesized, and evaluated for its dual inhibitory activities against MAO-A, MAO-B, and BACE1. Treatment of 4-nitrocinnamic acid with thionyl chloride resulted in the formation of acid chloride, and further reactions with selected substituted primary amines afforded the analogues (**NEA1** to **NEA5**). Among these compounds, **NEA3** and **NEA1** exerted more potent MAO-B activities (IC_50_ = 0.0092 and 0.016 μM, respectively) than the standard drugs (lazabemide and pargyline; IC_50_ = 0.11 and 0.14 μM, respectively). Moreover, these analogues exerted >1650-fold selectivity toward the MAO-B over MAO-A. A similar trend was observed in the case of BACE1 inhibition, wherein **NEA3** and **NEA1** exerted inhibitory activities with IC_50_ values of 8.01 and 8.21 μM, respectively. Enzyme kinetics of these analogues revealed a competitive and reversible inhibitory mode toward MAO-B. Molecular modeling studies further supported the ability of these analogues in their potential inhibitory activities. In summary, the present study identified the potent lead molecules, which will pave the way for new clinical agents for the treatment of neurodegenerative disorders.

## Figures and Tables

**Table 1:** MAOs and BACE1 inhibition profiles of nitro-bearing enamides ^a^.

Compounds	% Residual Activity (at 10 µM)			IC_50_ (µM)			SI ^b^
	MAO-A	MAO-B	BACE1	MAO-A	MAO-B	BACE1	
**NEA1**	91.9 ± 5.9	3.88 ± 0.6	34.9 ± 0.5	>40	0.016 ± 0.001	8.21 ± 0.03	>2500.0
**NEA2**	95.0 ± 0.5	35.8 ± 6.2	88.1 ± 0.3	>40	5.28 ± 0.09		>7.58
**NEA3**	61.9 ± 0.4	3.04 ± 0.5	34.4 ± 0.2	15.2 ± 0.6	0.0092 ± 0.0003	8.02 ± 0.13	>1652.2
**NEA4**	85.6 ± 1.7	9.52 ± 3.5	61.2 ± 3.0	>40	0.074 ± 0.020		>540.5
**NEA5**	83.5 ± 1.3	8.67 ± 2.3	44.0 ± 0.2	>40	0.038 ± 0.003	17.70 ± 1.70	>1052.6
Toloxatone				1.08 ± 0.03	-	-	
Lazabemide				-	0.11 ± 0.02	-	
Clorgyline				0.007 ± 0.001	-	-	
Pargyline				-	0.14 ± 0.01	-	
Quercetin				-	-	13.40 ± 0.04	

^a^ Results are the means ± S.E.M. of duplicate or triplicate experiments. ^b^ Selectivity index (SI) values are expressed for MAO-B as compared with MAO-A.

**Table 2:** BBB assay of enamide derivatives (**NEA1**–**NEA5**) using the PAMPA method.

Compounds	Bibliography [34] Pe (×10^−6^ cm/s)	Experimental Pe (×10^−6^ cm/s)	Prediction
**NEA1**		14.44 ± 0.35	CNS+
**NEA2**		13.46 ± 0.26	CNS+
**NEA3**		16.43 ± 0.17	CNS+
**NEA4**		15.54 ± 0.62	CNS+
**NEA5**		15.53 ± 0.44	CNS+
Progesterone	9.3	9.12 ± 0.21	CNS+
Verapamil	16.0	16.33 ± 0.44	CNS+
Piroxicam	2.5	2.37 ± 0.33	CNS±
Lomefloxacin	1.1	1.31 ± 0.51	CNS−
Dopamine	0.2	0.26 ± 0.04	CNS−

CNS+ (high BBB permeation predicted: Pe (10^−6^ cm/s) > 4.00). CNS− (low BBB permeation predicted: Pe (10^−6^ cm/s) < 2.00). CNS± (BBB permeation uncertain: Pe (10^−6^ cm/s) from 2.00 to 4.00).

## Data Availability

Not applicable.

## Supplementary Materials

The following are available online. ^1^H- and ^13^C-NMR spectral data of the compounds are available in the Supplementary Materials.

## Sample Availability

Samples of the compounds used in this study are available from the authors.
