# Supplementary material for: Selected Class of Enamides Bearing Nitro Functionality as Dual-Acting with Highly Selective Monoamine Oxidase-B and BACE1 Inhibitors
Source: Molecules. 2021 Oct 3;26(19):6004. doi: 10.3390/molecules26196004 (PMC8512054; doi:10.3390/molecules26196004)

Supplementary Information

Article

Selected Class of Enamides Bearing Nitro Pharmacophore as Dual-Acting with Highly Selective Monoamine Oxidase-B and BACE-1 Inhibitors

Anusree Venkidath [1,†], Jong Min Oh [2,†], Sanal Dev [1,*], Elham Amin [3,4], Shebina P. Rasheed [1], Ajeesh Vengamthodi [1], Nicola Gambacorta [5], Ahmed Khames [6], Mohamed A. Abdelgawad [7], Ginson George [8], Orazio Nicolotti [5], Hoon Kim [2,*] and Bijo Mathew [8,*]

[1] Centre for Experimental Drug Design and Development, Department of Pharmaceutical Chemistry, Al-Shifa College of Pharmacy, Perinthalmanna 679325, India; anusreekousthubham96@gmail.com (A.V.); shebinaniz@gmail.com (S.P.R.); ajeeshvengan@hotmail.com (A.V.)

[2] Department of Pharmacy, and Research Institute of Life Pharmaceutical Sciences, Sunchon National University, Suncheon 57922, Korea; 1205027@s.scnu.ac.kr

[3] Department of Medicinal Chemistry and Pharmacognosy, College of Pharmacy, Qassim University, Buraidah 52571, Saudi Arabia; elham_bns@yahoo.com (E.A.)

[4] Department of Pharmacognosy, Faculty of Pharmacy, Beni-Suef University, Beni-Suef 62514, Egypt

[5] Dipartimento di Farmacia-Scienze del Farmaco, Università degli Studi di Bari "Aldo Moro," via E. Orabona, 4, I-70125 Bari, Italy; nicola.gambacorta1@uniba.it (N.G.); orazio.nicolotti@uniba.it (O.N.)

[6] Department of Pharmaceutics and Industrial Pharmacy, College of Pharmacy, Taif University, P.O. Box-11099, Taif 21944, Saudi Arabia; a.khamies@tu.edu.sa

[7] Department of Pharmaceutical Chemistry, College of Pharmacy, Jouf University, Sakaka 72341, Saudi Arabia; mhmdgwd@ju.edu.sa

[8] Department of Pharmaceutical Chemistry, Amrita School of Pharmacy, Amrita Vishwa Vidyapeetham, AIMS Health Sciences Campus, Kochi 682 041, India; ginsongeorge239@gmail.com

* Correspondence: sanaldev@gmail.com (S.D.); hoon@sunchon.ac.kr (H.K.); bijovilaventgu@gmail.com or bijomathew@aims.amrita.edu (B.M.)

† These authors contributed equally.

3-(4-Nitrophenyl)-*N*-phenylacrylamide (NEA1)

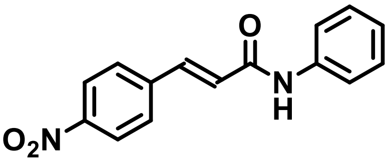

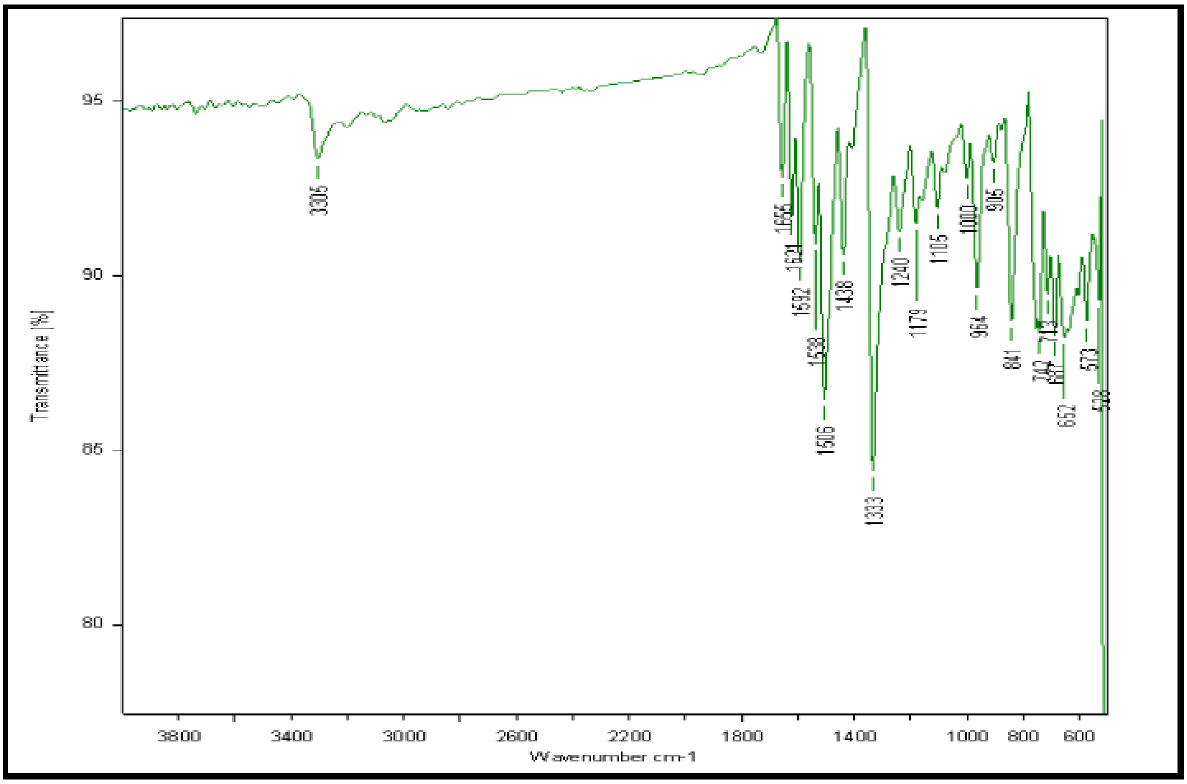

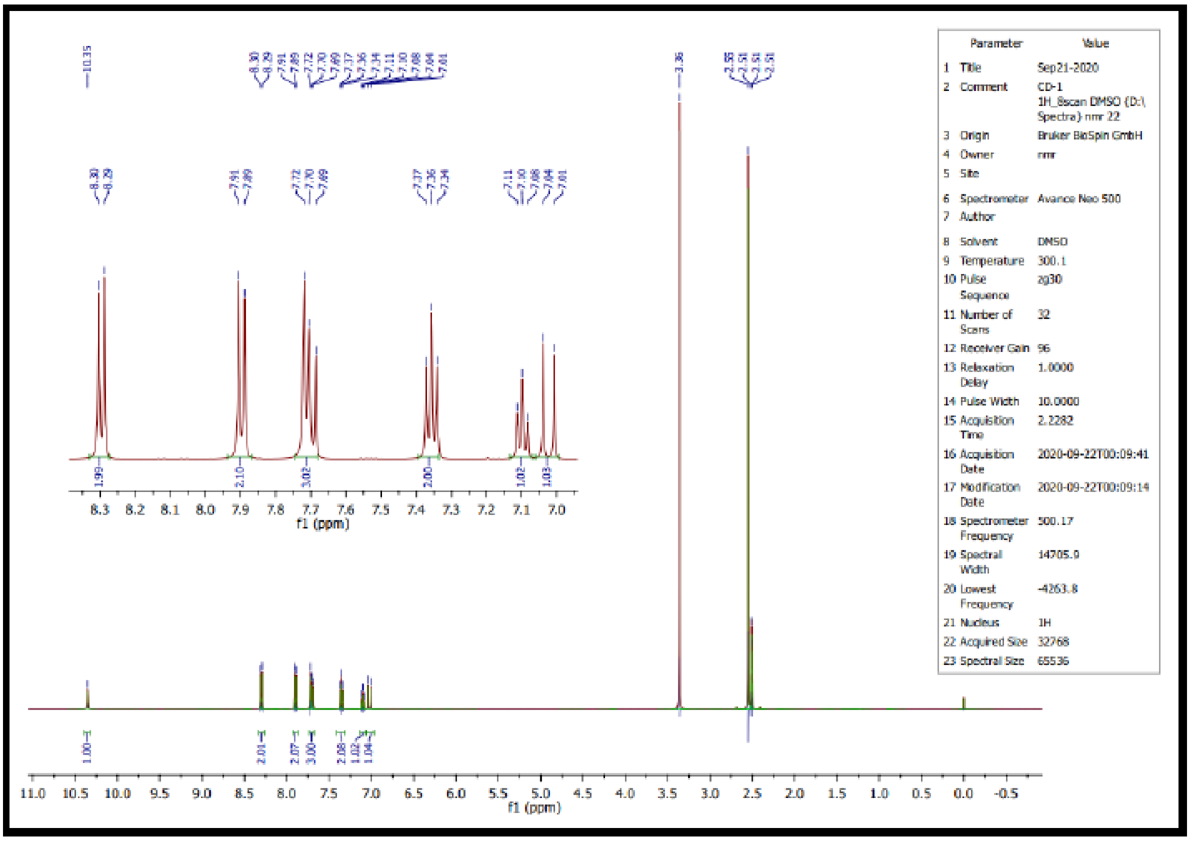

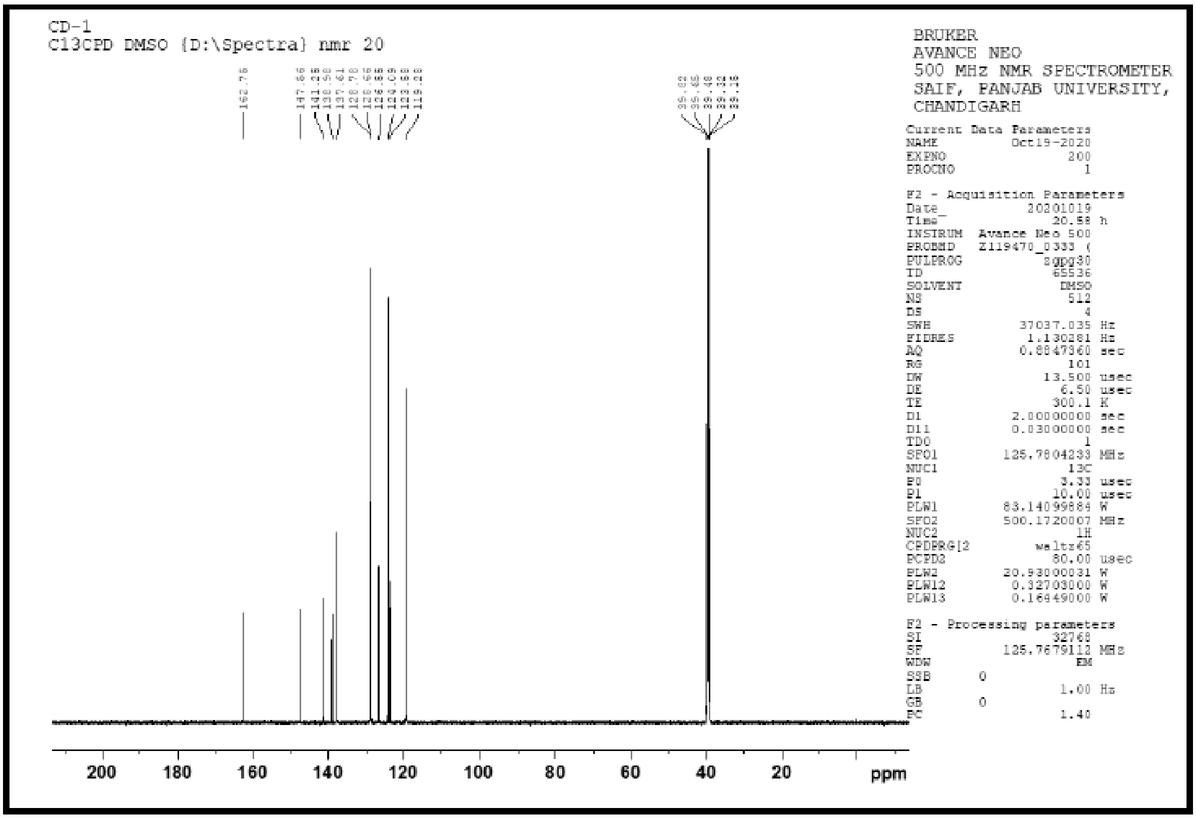
CD-1
C13CPD DMSO {D:\Spectra} nmr 20
BRUKER
AVANCE NEO
500 MHz NMR SPECTROMETER
SAIF, PANJAB UNIVERSITY,
CHANDIGARH
Current Data Parameters
NAME Oct19-2020
EXPNO 200
PROCNO 1
F2 - Acquisition Parameters
Date_ 20201019
Time 20.58 h
INSTRUM Avance Neo 500
PROBHD Z119470_0333 (
PULPROG zgpg30
TD 65536
SOLVENT DMSO
NS 512
DS 4
SWH 37037.035 Hz
FIDRES 1.130281 Hz
AQ 0.8847360 sec
RG 101
DW 13.500 usec
DE 6.50 usec
TE 300.1 K
D1 2.00000000 sec
D11 0.03000000 sec
TD0 1
SFO1 125.7804233 MHz
NUC1 13C
P0 3.33 usec
P1 10.00 usec
PLW1 83.14099884 W
SFO2 500.1720007 MHz
NUC2 1H
CPDPRG[2 waltz65
PCPD2 80.00 usec
PLW2 20.93000031 W
PLW12 0.32703000 W
PLW13 0.16449000 W
F2 - Processing parameters
SI 32768
SF 125.7679112 MHz
WDW EM
SSB 0
LB 1.00 Hz
GB 0
PC 1.40
200
180
160
140
120
100
80
60
40
20
ppm

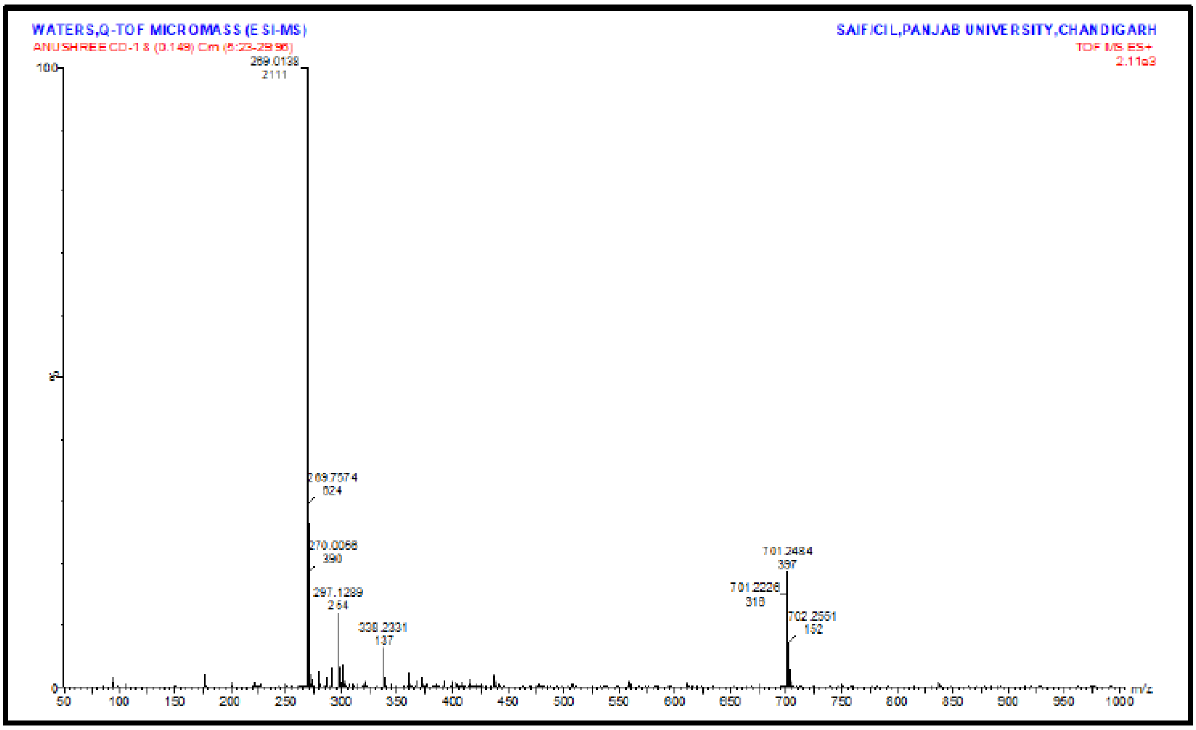
WATERS,Q-TOF MICROMASS (ESI-MS)
ANUSHREE CD-1 8 (0.149) Cm (5:23-29:95)
SAIF/CIL,PANJAB UNIVERSITY,CHANDIGARH
TOF MS ES+
2.11e3
269.0138
2111
269.7574
624
270.0068
390
297.1289
254
338.2331
137
701.2484
397
701.2226
318
702.2551
152
100
%
0
m/z

_N_-(4-Chlorophenyl)-3-(4-nitrophenyl)acrylamide (NEA2)

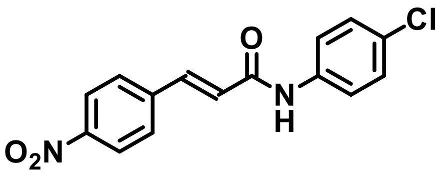

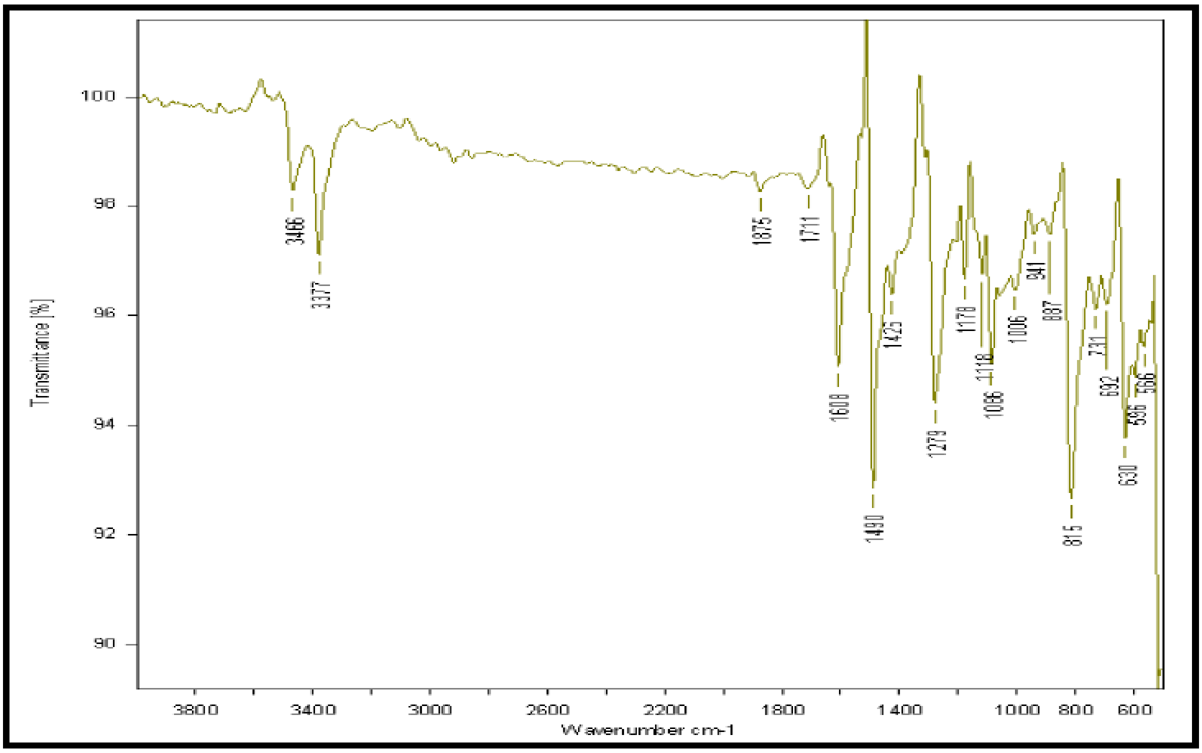

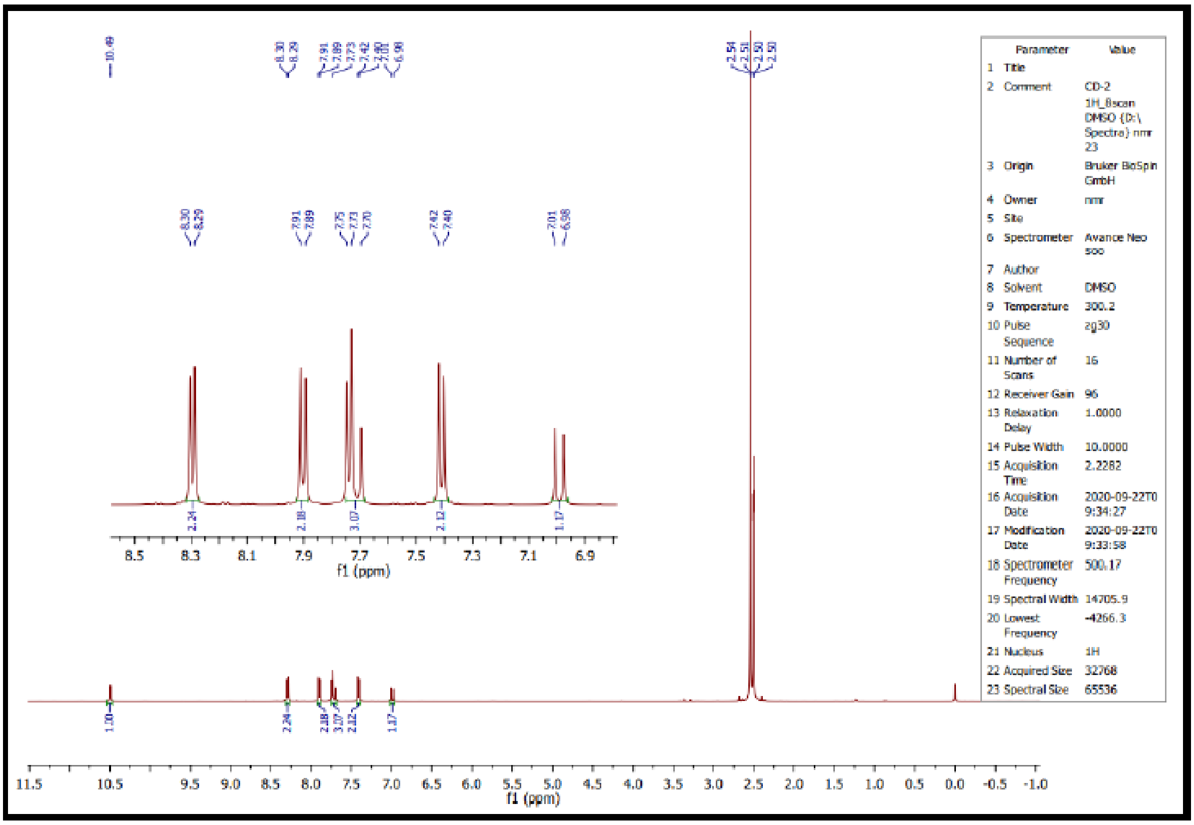

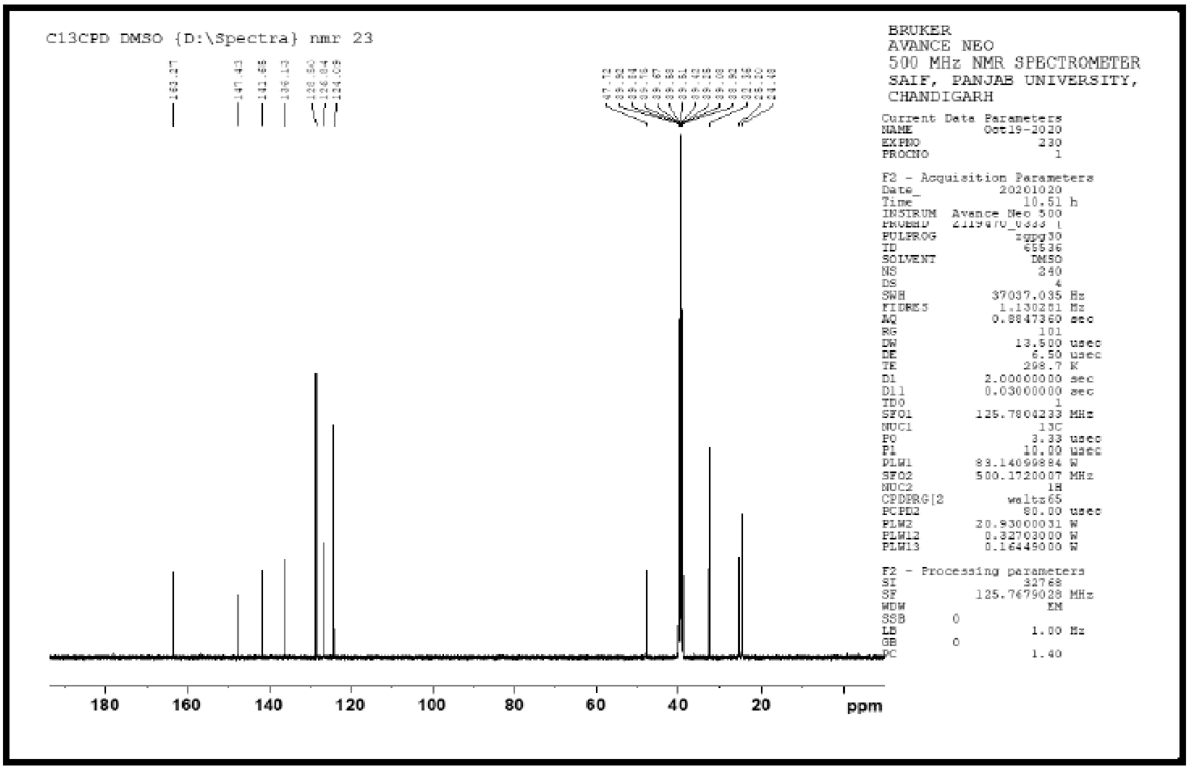

C13CPD DMSO {D:\Spectra} nmr 23
163.27
147.43
141.68
136.13
128.50
126.84
124.09
BRUKER
AVANCE NEO
500 MHz NMR SPECTROMETER
SAIF, PANJAB UNIVERSITY,
CHANDIGARH
Current Data Parameters
NAME Oct19-2020
EXPNO 230
PROCNO 1
F2 - Acquisition Parameters
Date_ 20201020
Time 10.51 h
INSTRUM Avance Neo 500
PULPROG zgpg30
TD 65536
SOLVENT DMSO
NS 240
DS 4
SWH 37037.035 Hz
FIDRES 1.130281 Hz
AQ 0.8847360 sec
RG 101
DW 13.500 usec
DE 6.50 usec
TE 298.7 K
D1 2.00000000 sec
D11 0.03000000 sec
TD0 1
SFO1 125.7804233 MHz
NUC1 13C
P0 3.33 usec
P1 10.00 usec
PLW1 83.14099884 W
SFO2 500.1720007 MHz
NUC2 1H
CPDPRG[2 waltz65
PCPD2 80.00 usec
PLW2 20.93000031 W
PLW12 0.32703000 W
PLW13 0.16449000 W
F2 - Processing parameters
SI 32768
SF 125.7679028 MHz
WDW EM
SSB 0
LB 1.00 Hz
GB 0
PC 1.40
180
160
140
120
100
80
60
40
20
ppm

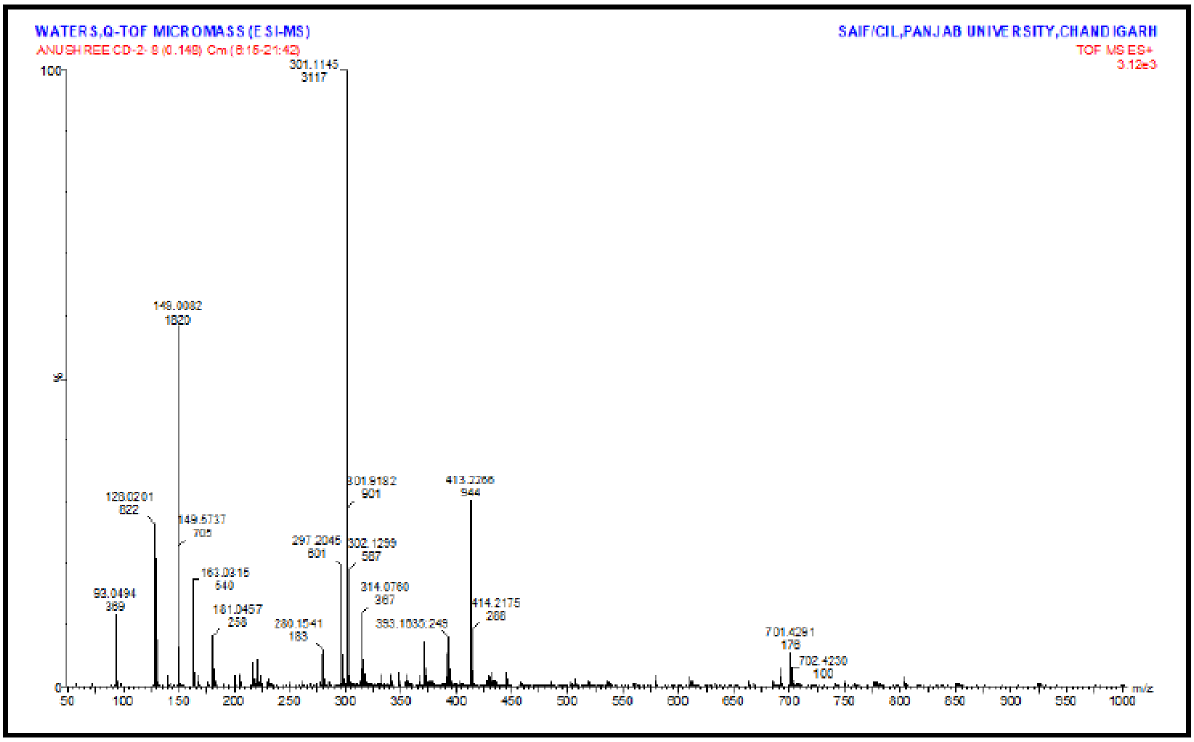

WATERS,Q-TOF MICROMASS (ESI-MS)
SAIF/CIL,PANJAB UNIVERSITY,CHANDIGARH
ANUSHREE CD-2- 8 (0.148) Cm (8:15-21:42)
TOF MS ES+
3.12e3
100
%
0
301.1145
3117
149.0082
1820
128.0201
822
149.5737
705
93.0494
389
163.0315
540
181.0457
258
280.1541
183
297.2045
801
301.9182
901
302.1299
587
314.0760
367
393.1030 248
413.2266
944
414.2175
288
701.4291
178
702.4230
100
m/z
50
100
150
200
250
300
350
400
450
500
550
600
650
700
750
800
850
900
950
1000

N-(4-Fluorophenyl)-3-(4-nitrophenyl)acrylamide (NEA3)

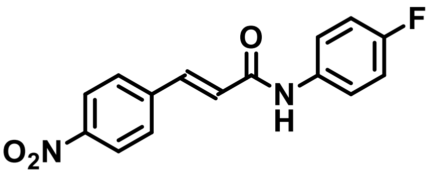

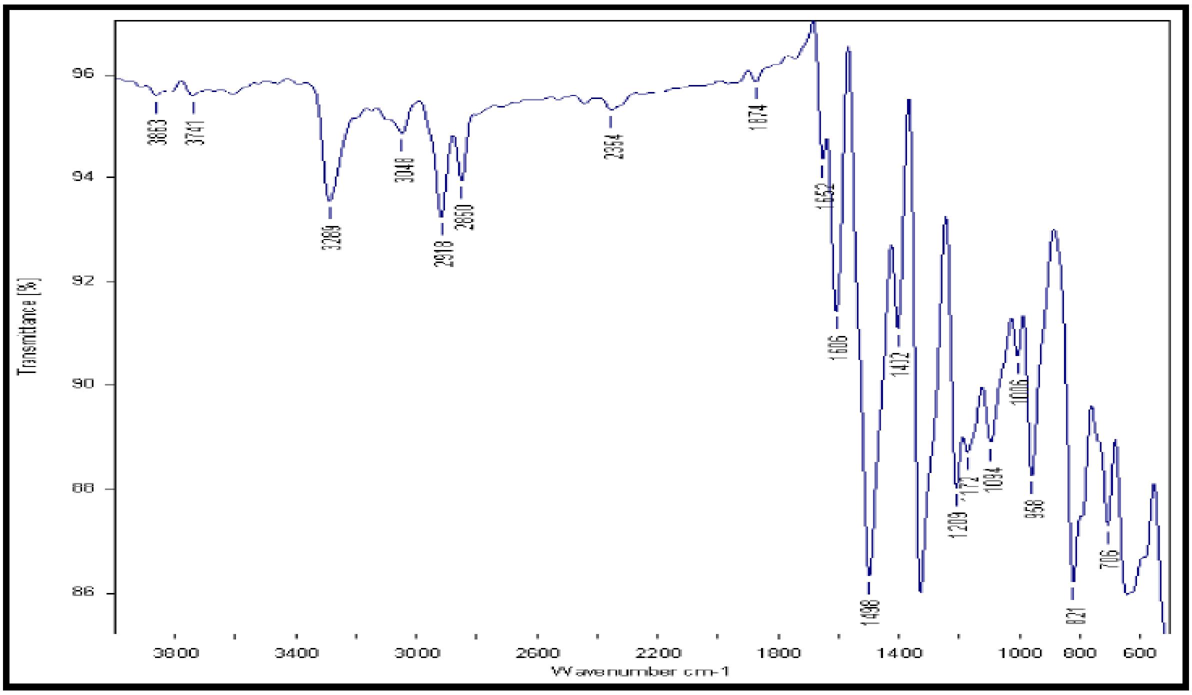

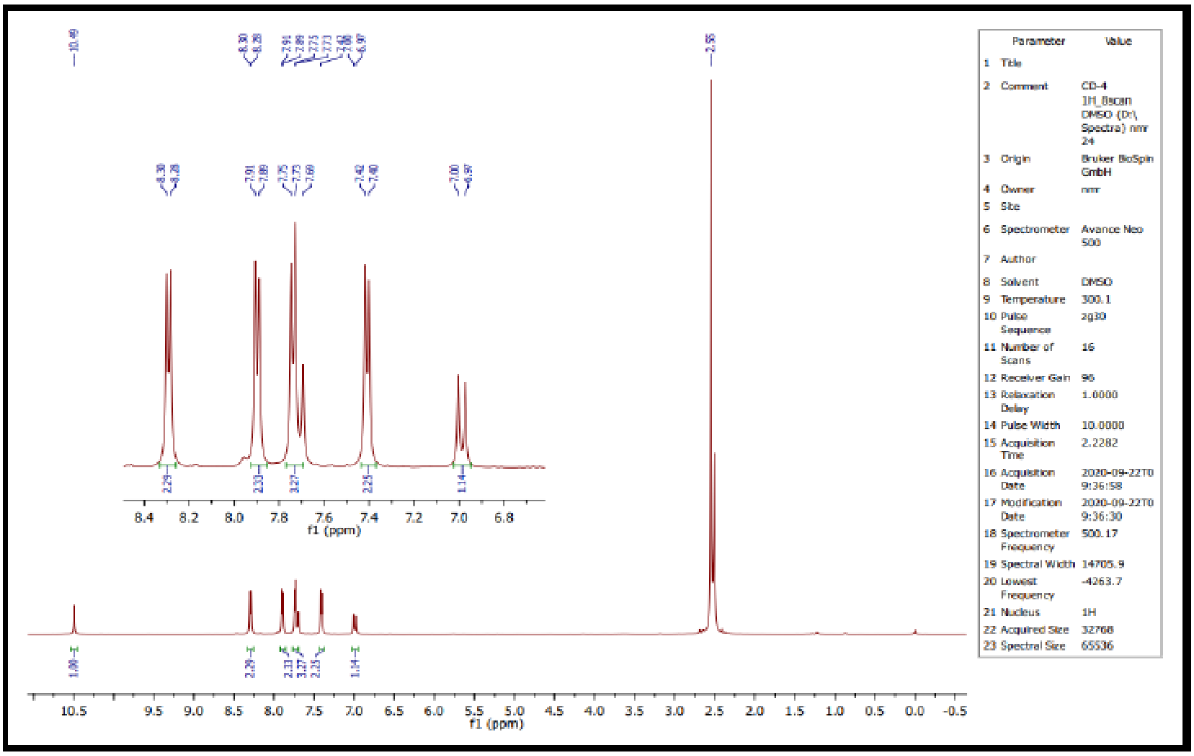

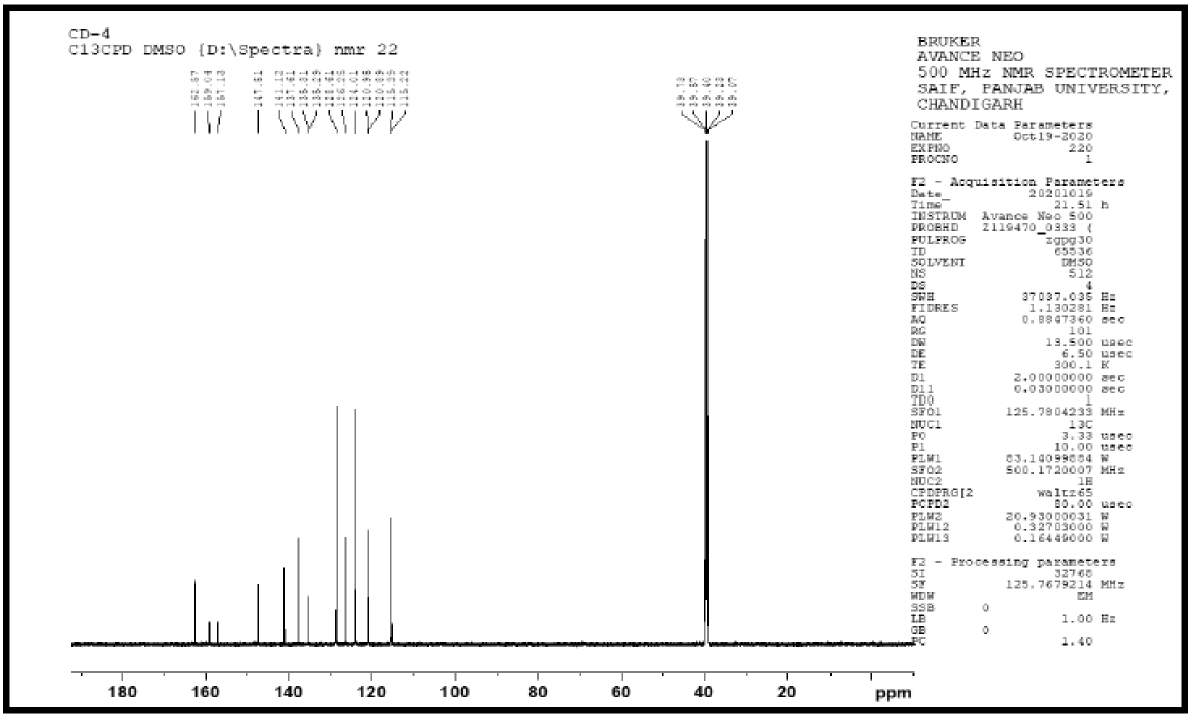
CD-4
C13CPD DMSO {D:\Spectra} nmr 22
BRUKER
AVANCE NEO
500 MHz NMR SPECTROMETER
SAIF, PANJAB UNIVERSITY,
CHANDIGARH
ppm

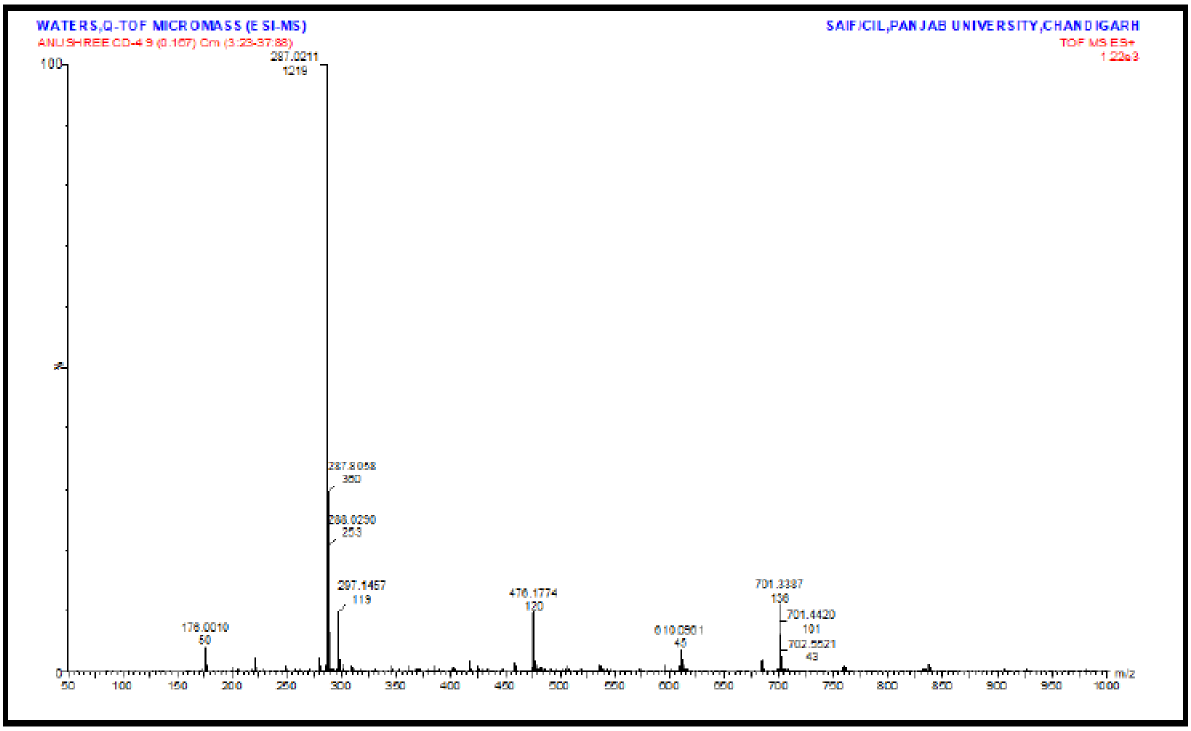
WATERS,Q-TOF MICROMASS (ESI-MS)
SAIF/CIL,PANJAB UNIVERSITY,CHANDIGARH
TOF MS ES+
287.0211
1219
m/z

***N*-Cyclohexyl-3-(4-nitrophenyl)acrylamide (NEA4)**

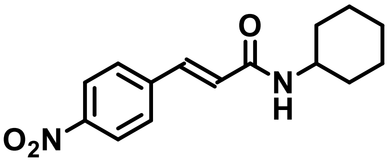

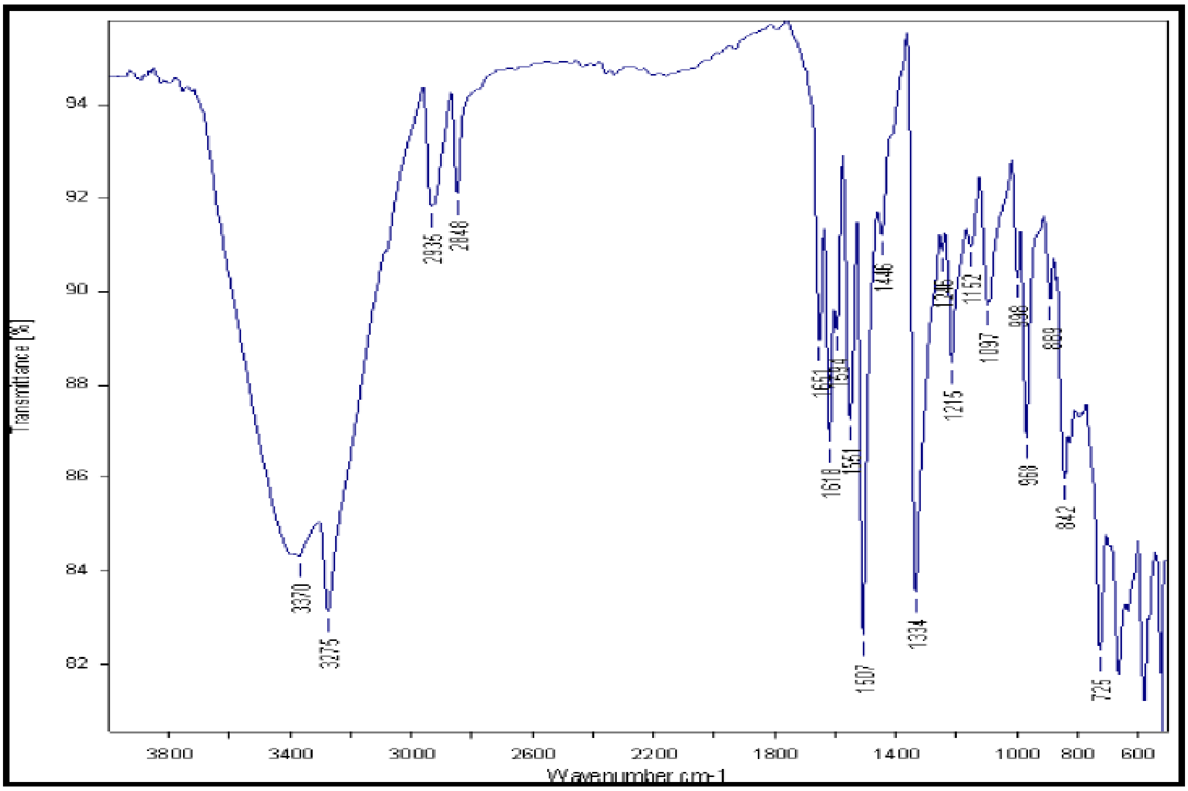

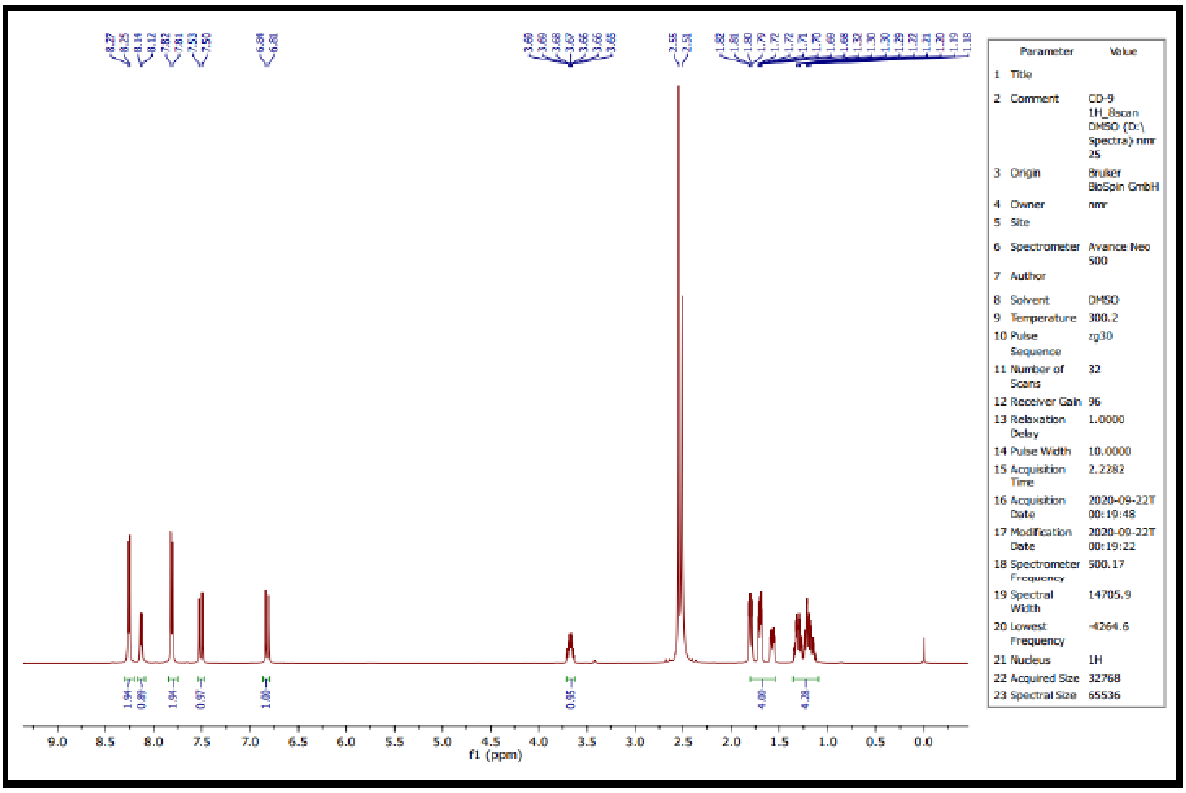

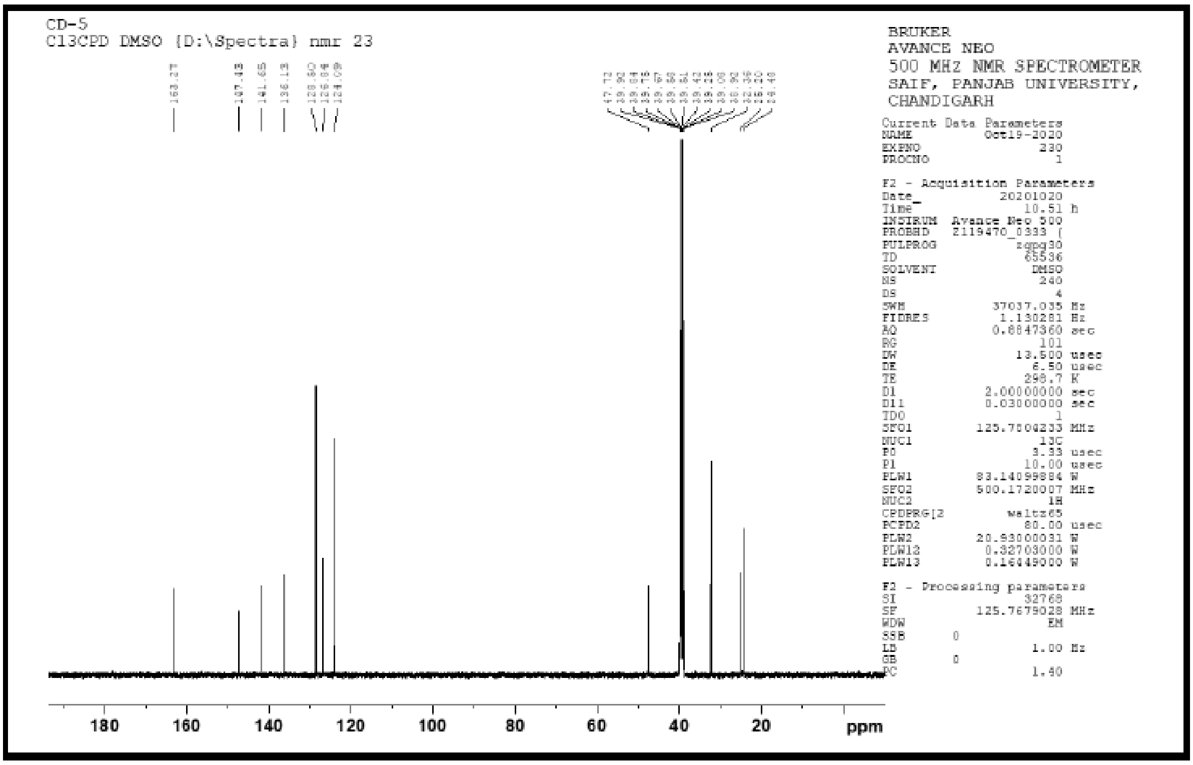

CD-5
C13CPD DMSO {D:\Spectra} nmr 23
163.27
147.43
141.65
136.13
128.80
126.84
124.09
BRUKER
AVANCE NEO
500 MHz NMR SPECTROMETER
SAIF, PANJAB UNIVERSITY,
CHANDIGARH
Current Data Parameters
NAME Oct19-2020
EXPNO 230
PROCNO 1
F2 - Acquisition Parameters
Date_ 20201020
Time 10.51 h
INSTRUM Avance Neo 500
PROBHD Z119470_0333 (
PULPROG zgpg30
TD 65536
SOLVENT DMSO
NS 240
DS 4
SWH 37037.035 Hz
FIDRES 1.130281 Hz
AQ 0.8847360 sec
RG 101
DW 13.500 usec
DE 6.50 usec
TE 298.7 K
D1 2.00000000 sec
D11 0.03000000 sec
TD0 1
SFO1 125.7804233 MHz
NUC1 13C
P0 3.33 usec
P1 10.00 usec
PLW1 83.14099884 W
SFO2 500.1720007 MHz
NUC2 1H
CPDPRG[2 waltz65
PCPD2 80.00 usec
PLW2 20.93000031 W
PLW12 0.32703000 W
PLW13 0.16449000 W
F2 - Processing parameters
SI 32768
SF 125.7679028 MHz
WDW EM
SSB 0
LB 1.00 Hz
GB 0
PC 1.40
180
160
140
120
100
80
60
40
20
ppm

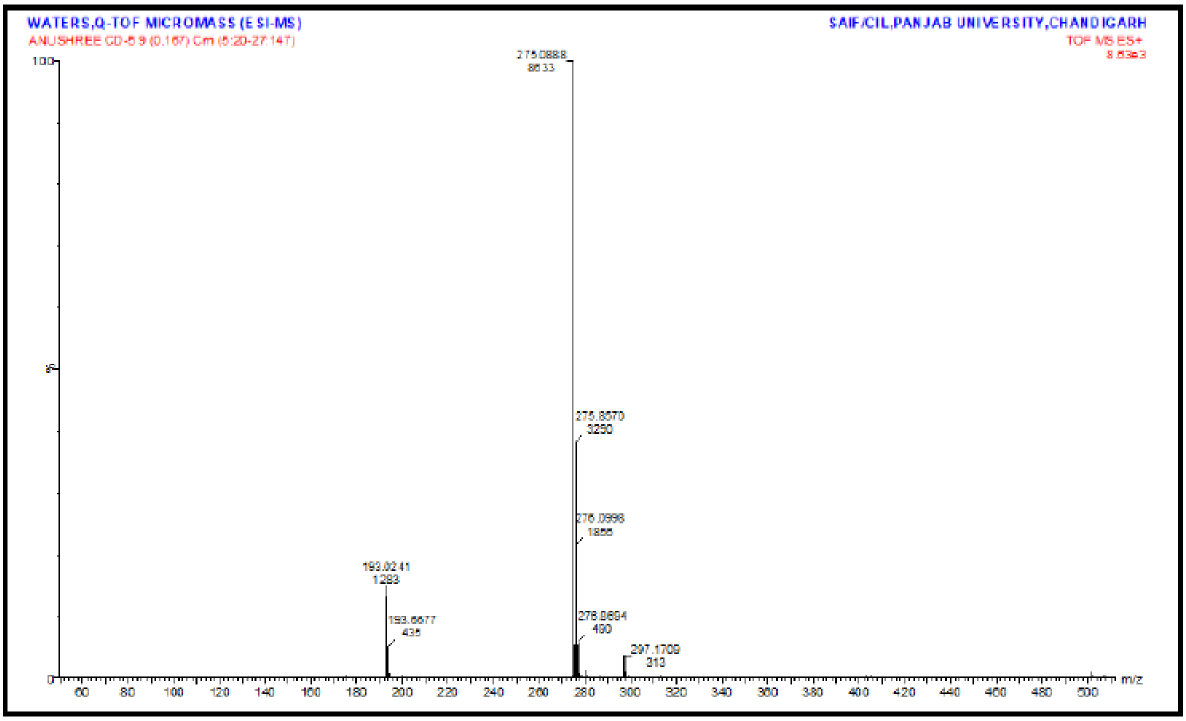

WATERS,Q-TOF MICROMASS (ESI-MS)
SAIF/CIL,PANJAB UNIVERSITY,CHANDIGARH
TOF MS ES+
100
%
0
275.0888
276.0998
193.0241
297.1709
m/z

3-(4-Nitrophenyl)-N-(o-tolyl)acrylamide (NEA5)

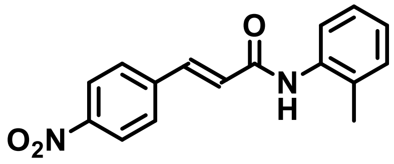

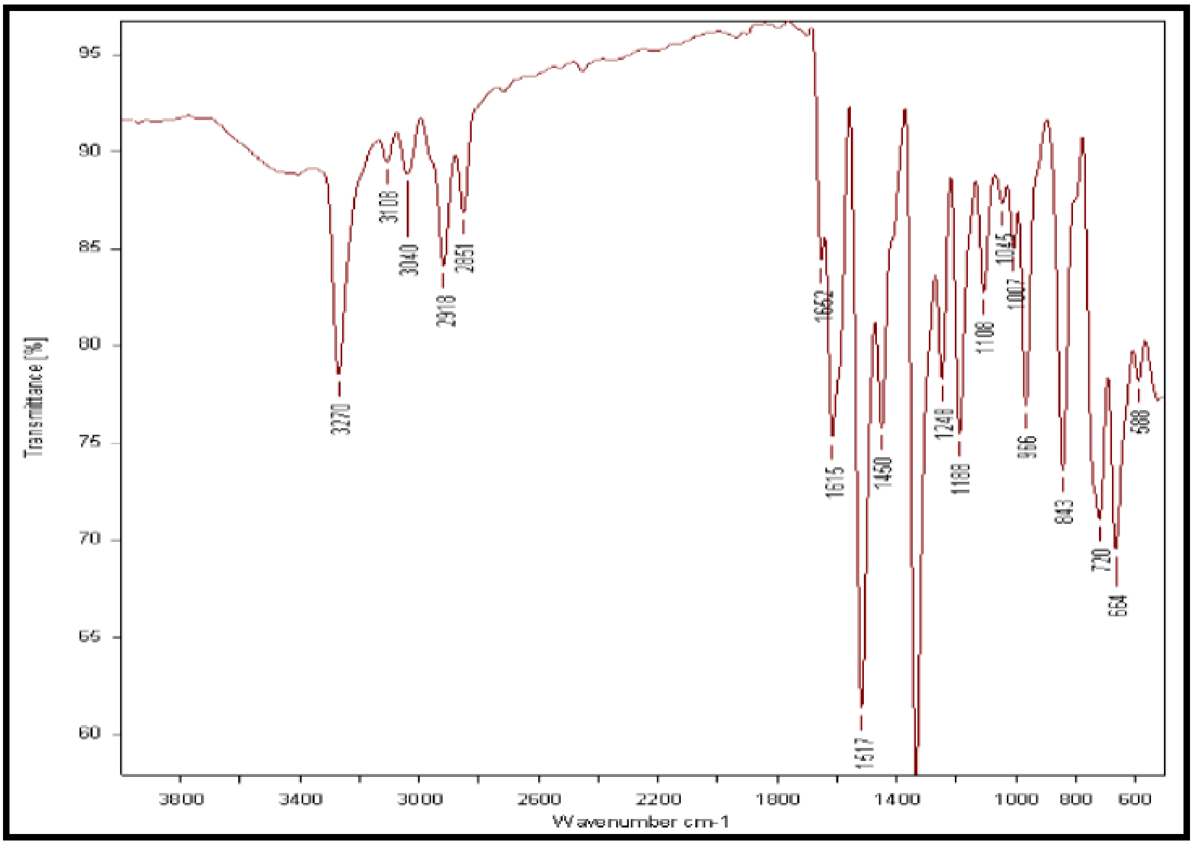

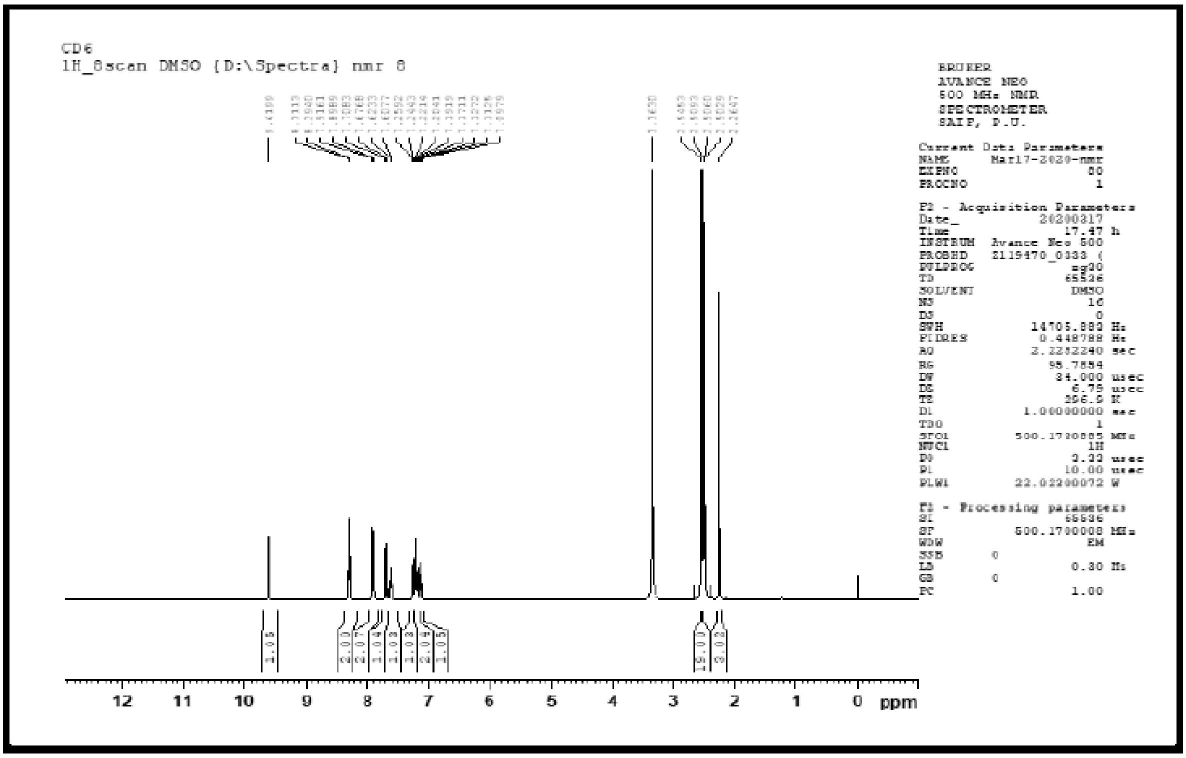

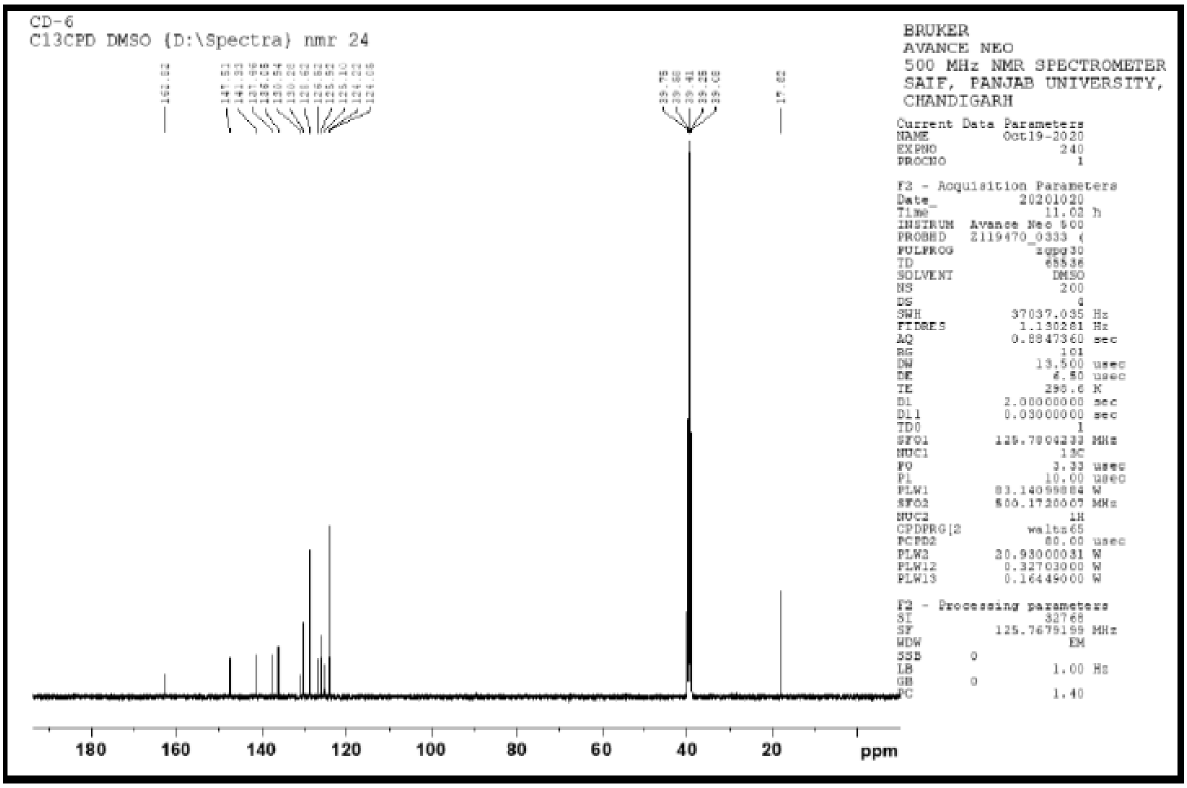
CD-6
C13CPD DMSO (D:\Spectra) nmr 24
BRUKER
AVANCE NEO
500 MHz NMR SPECTROMETER
SAIF, PANJAB UNIVERSITY,
CHANDIGARH
Current Data Parameters
NAME Oct19-2020
EXPNO 240
PROCNO 1
F2 - Acquisition Parameters
Date_ 20201020
Time 11.02 h
INSTRUM Avance Neo 500
PROBHD Z119470_0333 (
SOLVENT DMSO
NS 200
DS 4
SWH 37037.035 Hz
FIDRES 1.130281 Hz
AQ 0.8847360 sec
RG 101
DW 13.500 usec
D1 2.00000000 sec
D11 0.03000000 sec
TD0 1
P1 10.00 usec
NUC2 1H
CPDPRG[2 waltz65
PLW2 20.93000031 W
PLW12 0.32703000 W
PLW13 0.16449000 W
F2 - Processing parameters
SI 32768
SF 125.7679199 MHz
WDW EM
SSB 0
LB 1.00 Hz
GB 0
PC 1.40
180
160
140
120
100
80
60
40
20
ppm

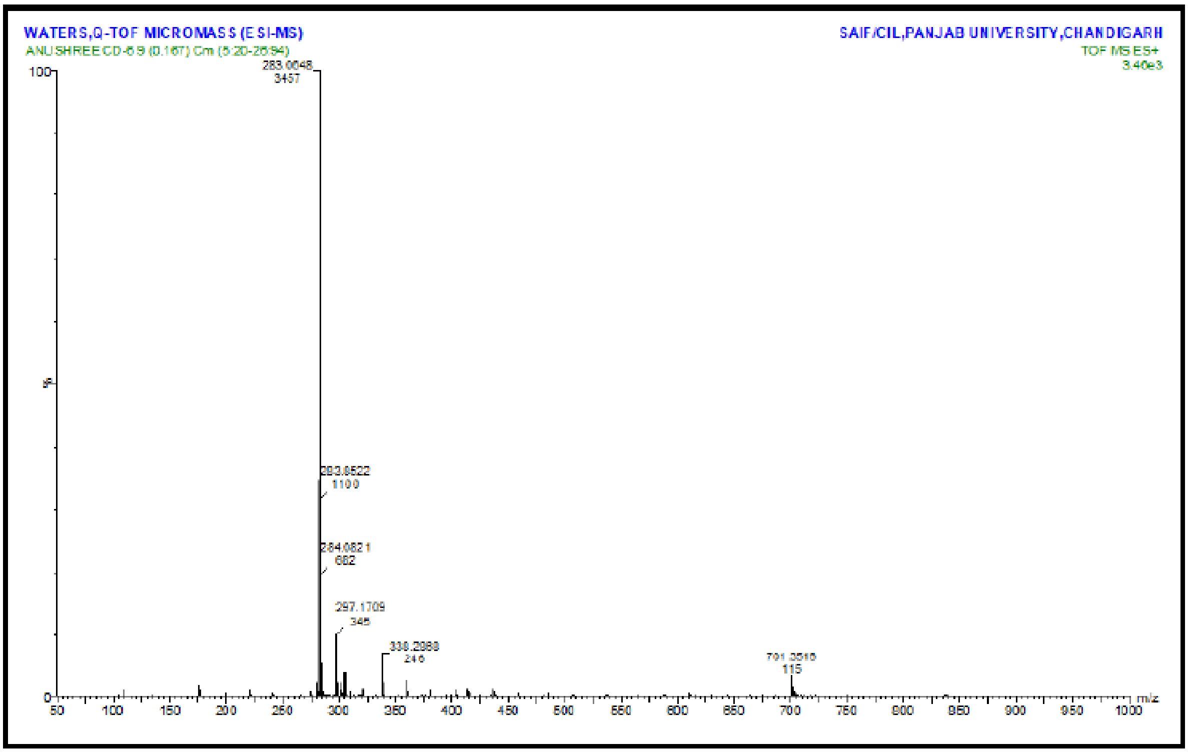
WATERS,Q-TOF MICROMASS (ESI-MS)
ANUSHREE CD-6 9 (0.167) Cm (5:20-26:94)
SAIF/CIL,PANJAB UNIVERSITY,CHANDIGARH
TOF MS ES+
3.40e3
100
%
0
283.0048
3457
283.8522
1100
284.0821
682
297.1709
345
338.2968
246
701.3516
115
50
100
150
200
250
300
350
400
450
500
550
600
650
700
750
800
850
900
950
1000
m/z

EXPANDED 1H-NMR of NEA4

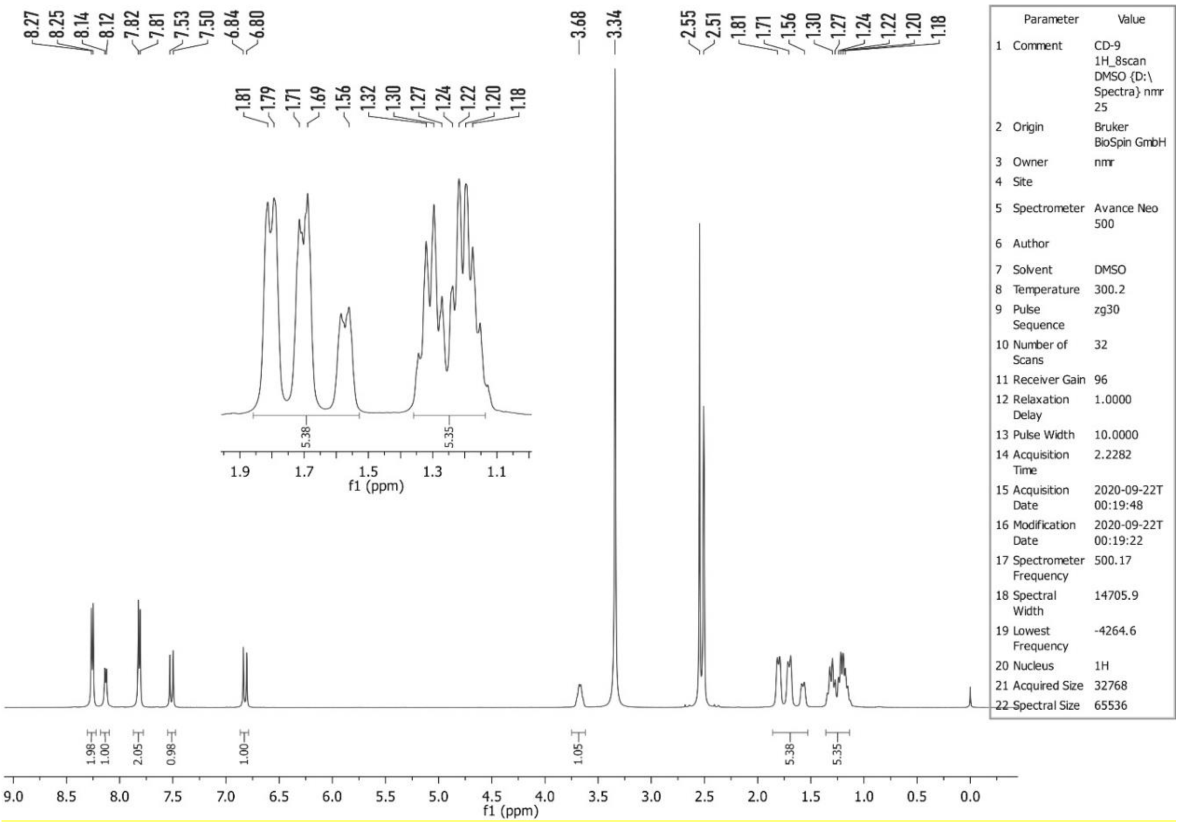

EXPANDED 1H-NMR of NEA5

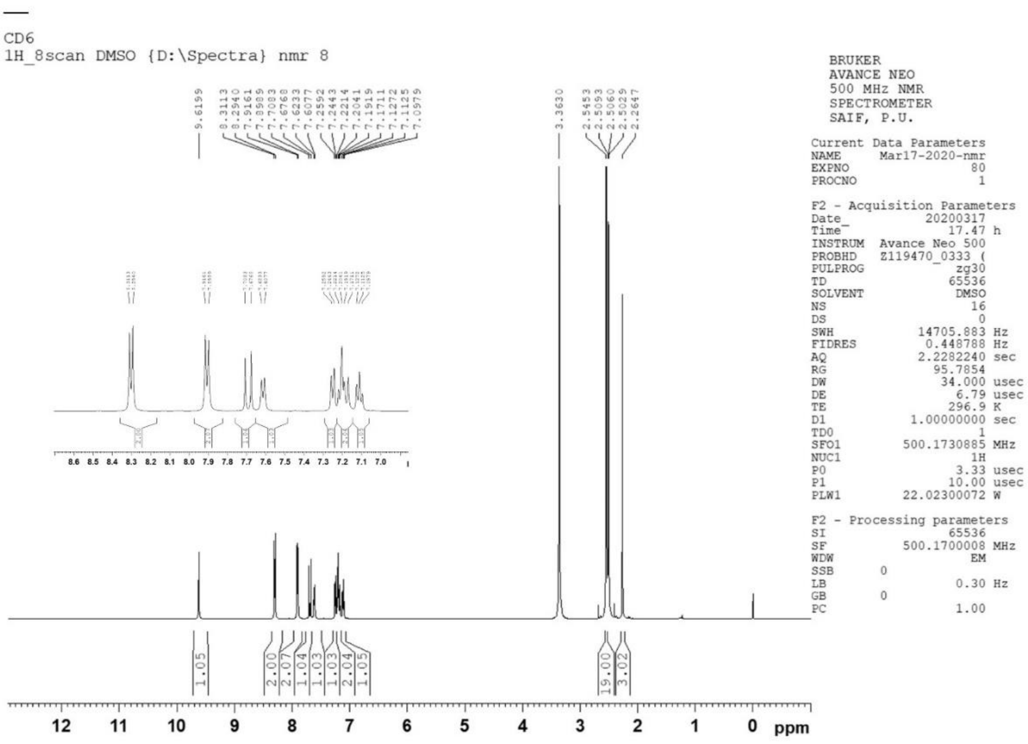

Supplement: Supplementary file 1 [file molecules-26-06004-s001.zip › molecules-1309142-supplementary.pdf]
